# Introducing an algal carbon‐concentrating mechanism into higher plants: location and incorporation of key components

**DOI:** 10.1111/pbi.12497

**Published:** 2015-11-05

**Authors:** Nicky Atkinson, Doreen Feike, Luke C. M. Mackinder, Moritz T. Meyer, Howard Griffiths, Martin C. Jonikas, Alison M. Smith, Alistair J. McCormick

**Affiliations:** ^1^ SynthSys & Institute of Molecular Plant Sciences School of Biological Sciences University of Edinburgh Edinburgh UK; ^2^ Department of Metabolic Biology John Innes Centre Norwich Research Park Norwich UK; ^3^ Department of Plant Biology Carnegie Institution for Science Stanford CA USA; ^4^ Department of Plant Sciences University of Cambridge Cambridge UK

**Keywords:** photosynthesis improvement, carbon‐concentrating mechanism, bicarbonate transporter, *Arabidopsis thaliana*, tobacco, *Chlamydomonas reinhardtii*

## Abstract

Many eukaryotic green algae possess biophysical carbon‐concentrating mechanisms (CCMs) that enhance photosynthetic efficiency and thus permit high growth rates at low CO_2_ concentrations. They are thus an attractive option for improving productivity in higher plants. In this study, the intracellular locations of ten CCM components in the unicellular green alga *Chlamydomonas reinhardtii* were confirmed. When expressed in tobacco, all of these components except chloroplastic carbonic anhydrases CAH3 and CAH6 had the same intracellular locations as in Chlamydomonas. CAH6 could be directed to the chloroplast by fusion to an Arabidopsis chloroplast transit peptide. Similarly, the putative inorganic carbon (Ci) transporter LCI1 was directed to the chloroplast from its native location on the plasma membrane. CCP1 and CCP2 proteins, putative Ci transporters previously reported to be in the chloroplast envelope, localized to mitochondria in both Chlamydomonas and tobacco, suggesting that the algal CCM model requires expansion to include a role for mitochondria. For the Ci transporters LCIA and HLA3, membrane location and Ci transport capacity were confirmed by heterologous expression and H^14^ CO_3_^‐^ uptake assays in Xenopus oocytes. Both were expressed in Arabidopsis resulting in growth comparable with that of wild‐type plants. We conclude that CCM components from Chlamydomonas can be expressed both transiently (in tobacco) and stably (in Arabidopsis) and retargeted to appropriate locations in higher plant cells. As expression of individual Ci transporters did not enhance Arabidopsis growth, stacking of further CCM components will probably be required to achieve a significant increase in photosynthetic efficiency in this species.

## Introduction

Most plants, including the major grain crops rice and wheat, assimilate carbon using the C_3_ photosynthetic pathway. C_3_ plants rely on passive diffusion to deliver carbon dioxide (CO_2_) from the atmosphere (*ca*. 400 ppm) to the chloroplasts inside leaf mesophyll cells, wherein CO_2_ photoassimilation proceeds via the primary carboxylase enzyme, ribulose‐1,5‐bisphosphate carboxylase/oxygenase (RuBisCO, EC 4.1.1.39). Diffusive resistances result in a gradient (*ca*. 40% under high irradiance) between CO_2_ levels in the substomatal cavity of the leaf and the steady‐state level of dissolved CO_2_ in chloroplasts (10–20 μm at 25 °C) (Price *et al*., 2011). Here, CO_2_ is not saturating for RuBisCO and oxygen (O_2_; *ca*. 250 μm at 25 °C) competes at the RuBisCO active sites, resulting in both loss of assimilated carbon and nitrogen and energy consumption in the photorespiratory pathway that recycles the product of RuBP oxygenation (Sharkey, 1988). The productivity of C_3_ crops is thus limited by the efficiency of CO_2_ photoassimilation, even when grown under elevated CO_2_ levels (up to 650 ppm) (Long *et al*., 2006). Generating C_3_ crop plants with increased photosynthetic efficiencies is a major target for improving yields and safeguarding future food security. Strategies under consideration and development include modifying canopies to increase light interception, enhancing repair mechanisms to overcome lags associated with photoprotection, increasing the efficiency of RuBisCO and eliminating photorespiration by introducing molecular components of microbial carbon‐concentrating mechanisms (CCM) (Lin *et al*., 2014a,b; Long *et al*., 2015; Parry *et al*., 2013; Whitney *et al*., 2011; Zhu *et al*., 2010).

Many photosynthetic organisms including cyanobacteria, most green algae and a single group of land plants, the hornworts, have evolved biophysical CCMs that actively increase the CO_2_ concentration around RuBisCO, thus suppressing RuBisCO oxygenase activity and associated photorespiration. In eukaryotic algae, CCMs involve inorganic carbon (Ci) transporters at the plasma membrane and chloroplast envelope and carbonic anhydrases, which act in concert to deliver above ambient concentrations of CO_2_ to RuBisCO, usually within a chloroplast microcompartment called the pyrenoid. The pyrenoid is mainly composed of densely packaged RuBisCO (Engel *et al*., 2015). Whilst not all algae with a CCM have a pyrenoid, the microcompartment enhances the efficiency of CO_2_ assimilation (Morita *et al*., 1998). Theoretical modelling approaches have demonstrated the requirement for a pyrenoid in algal systems (Badger *et al*., 1998) and shown that some form of microcompartment containing RuBisCO would also be needed for a successful CCM in higher plant systems (Price *et al*., 2013).

The best‐characterized algal CCM is that of the model green alga *Chlamydomonas reinhardtii* (Chlamydomonas throughout). To date, a large number of molecular components have been implicated in the Chlamydomonas CCM through mutant screens, transcriptomic studies and functional homology with components in other photosynthetic organisms (Brueggeman *et al*., 2012; Fang *et al*., 2012; Meyer and Griffiths, 2013). At least 14 genes are thought to be important in maintaining a fully functional CCM under ambient or below ambient CO_2_ concentrations (see Wang *et al*. (2015) for review). Briefly, these include five Ci transporters, four CAs, two pyrenoid peripheral proteins, a putative methyl transferase and two nuclear transcription regulators. The precise function and importance of these components remains only partly understood. In addition, the Chlamydomonas CCM requires a RuBisCO that can be targeted to the pyrenoid, a property known to be dependent on sequence elements of the RuBisCO small subunit (Genkov *et al*., 2010; Meyer *et al*., 2012).

To examine the feasibility of enhancing photosynthetic efficiency in C_3_ plants by introducing CCM components from Chlamydomonas, we chose ten components that are considered essential or potentially important for CCM functionality (Table 1). These included intracellular components that mediate the transport and conversion of Ci from the external environment to the active sites of RuBisCO. LCI1 and HLA3 are putative Ci transporters reportedly in the plasma membrane; LCIA, CCP1 and CCP2 are putative chloroplast envelope Ci transporters, although only the former has been localized *in vivo*. CAH1, CAH3 and CAH6 are carbonic anhydrases, thought to be located in the periplasmic space (CAH1), the thylakoid lumen (CAH3) and the chloroplast stroma (CAH6). LCIB and LCIC are proteins of unclear function that are reported to surround the pyrenoid when CO_2_ becomes limiting.

**Table 1:** Chlamydomonas CCM genes used in this study. Locus name refers to the gene ID as supplied by Phytozome v5.5 (http://phytozome.jgi.doe.gov/pz/portal.html#!info?alias=Org_Creinhardtii)

Gene	Locus name	Protein length, size of precursor	Putative function in native algal CCM	Examples of experimental evidence for function	Mutant phenotype
*HLA3*	Cre02.g097800	1325 aa, 147 kDa	Ci uptake into cytosol	RNAi lines grown under alkaline conditions (main Ci species is ${\text{HCO}}_{3}^{-}$) have a HCR (1); synergistic effect with LCIA (1;2); ^14^Ci uptake assay (2;3); affinity for Ci when controlling gene expression with dTALE (3); + work presented here	Reduction in Ci accumulation when CO_2_ <0.02%; HCR
*LCI1*	Cre03.g162800	192 aa, 21 kDa	Ci uptake into cytosol	^14^Ci uptake assay (4)	No published mutant; overexpression in CCM regulatory mutant promotes ${\text{HCO}}_{3}^{-}$ uptake
*LCIA*	Cre06.g309000	336 aa, 35 kDa	Ci transport from cytosol to stroma	Synergistic effect with HLA3 (2;3); electrophysiology in Xenopus oocytes (5); reduced affinity for Ci at alkaline pH (6); + work presented here	Reduction in Ci accumulation when CO_2_ <0.02%; HCR
*CCP1*	Cre04.g223300	358 aa, 38 kDa	Ci transport from cytosol to stroma	RNAi lines support role in Ci transport (7); putative localization inferred bioinformatically	No published mutant
*CCP2*	Cre04.g222750	355 aa, 38 kDa	Ci transport from cytosol to stroma	RNAi lines support role in Ci transport (7); putative localization inferred bioinformatically	No published mutant
*CAH1*	Cre04.g223100	377 aa, 42 kDa	CO_2_ and ${\text{HCO}}_{3}^{-}$ at cell surface	Absence of growth effect in the presence of membrane impermeable CA inhibitors (9)	No apparent deleterious effect on growth
*CAH6*	Cre12.g485050	264 aa, 28 kDa	Recapture of CO_2_ leaking from the pyrenoid	No experimental evidence of function; putative role in CCM inferred from putative localization	No published mutant
*CAH3*	Cre09.g415700	310 aa, 33 kDa	Terminal dehydration of ${\text{HCO}}_{3}^{-}$ to CO_2,_ to saturate RuBisCO in the pyrenoid	Low CO_2_‐induced phosphorylation relocalizes CAH3 preferentially to pyrenoid tubules (13); alternative CCM‐unrelated function (regulation of water oxydation at PSII) has been proposed (14)	Mutation produces overaccumulation of Ci
*LCIB*	Cre10.g452800	448 aa, 48 kDa	CO_2_ uptake or trapping of stromal CO_2_, pyrenoid localization	Synergetic role with Ci pumps (l*cia/lciB* double mutant lethal when CO_2_ <0.02%) (1); MS‐identification of LCIB‐FLAG pull‐down and gel filtration showed that LCIB‐C form a 360 kDa hetero‐hexamer, which localizes around the pyrenoid when CO_2_ <0.02% (16)	Lethal under air‐level CO_2_ but rescued when CO_2_ <0.02%
*LCIC*	Cre06.g307500	443 aa, 48 kDa	CO_2_ uptake or trapping of stromal CO_2_, pyrenoid localization	MS‐identification of LCIB‐FLAG pull‐down and gel filtration showed that LCIB‐C form a 360 kDa hetero‐hexamer, which localizes around the pyrenoid when CO_2_ <0.02% (16)	No published mutant

Gene	Locus name	Functional annotation	Location as per latest CCM model (Wang *et al*., 2015)	Examples of experimental evidence for location; work presented here for all ten genes	References
*HLA3*	Cre02.g097800	ABC transporter superfamily	Plasmamembrane	Immunofluorescence (2); immunoblot on membrane fractions (2;3)	(1) Duanmu *et al*. (2009a); (2) Yamano *et al*. (2015); (3) Gao *et al*. (2015)
*LCI1*	Cre03.g162800	Unknown	Plasmamembrane	Immunofluorescence + GFP fusion + immunoblot on membrane fractions (4)	(4) Ohnishi *et al*. (2010
*LCIA*	Cre06.g309000	Formate/nitrite transporter	Chloroplast membrane	Immunofluorescence (2;6)	(5) (Mariscal *et al*. (2006); (6) Wang and Spalding, (2014a)
*CCP1*	Cre04.g223300	Mitochondrial carrier protein	Chloroplast membrane	Immunoblot on membrane fractions (8)); + work presented here	(7) Pollock *et al*. (2004); (8) Ramazanov *et al*. (1993)
*CCP2*	Cre04.g222750	Mitochondrial carrier protein	Chloroplast membrane	Immunoblot on membrane fractions (8); + work presented here	(7) Pollock *et al*. (2004); (8) Ramazanov *et al*. (1993)
*CAH1*	Cre04.g223100	α carbonic anhydrase	Periplasmic space	Cell wall lysis + CA assay on supernatant (10); immunogold (11)	(9) Moroney and Tolbert, (1985); (10) Kimpel *et al*. (1983); (11) Ynalvez *et al*. (2008)
*CAH6*	Cre12.g485050	β carbonic anhydrase	Stroma	Immunogold (12)	(12) Mitra *et al*. (2004)
*CAH3*	Cre09.g415700	α carbonic anhydrase	Thylakoid lumen	Immunogold (12;15)	(13) Blanco‐Rivero *et al*. (2012); (14) Villarejo *et al*. (2002); (15) Markelova *et al*. (2009)
*LCIB*	Cre10.g452800	Unknown	Stroma	Immunogold (16); GFP fusion (16,17); immunofluorescence (6;18)	(16) Yamano *et al*. (2010); (17) Yamano *et al*. (2014); (18) Wang and Spalding (2014b)
*LCIC*	Cre06.g307500	Unknown	Stroma	Immunogold + immunofluorescence (16)	

CA, carbonic anhydrase; Ci, inorganic carbon; HCR: high CO_2_‐requiring phenotype, a nonlethal mutation rescued by growth under elevated CO_2_ (2–5% [v/v]).

We first expressed fluorescently tagged versions of all these proteins in Chlamydomonas cells to obtain definitive information about their locations and then expressed them transiently in tobacco (*Nicotiana benthamiana* L.) leaves. With one exception, proteins localized to identical compartments in the two organisms. Subsequent analyses focussed on the putative Ci transporters LCIA and HLA3, which have been shown to cooperatively drive bicarbonate uptake from the extracellular environment to the chloroplast stroma (Yamano *et al*., 2015). We showed that both function as Ci transporters when expressed in the outer membrane of Xenopus oocytes. We also expressed these proteins stably in transgenic Arabidopsis plants, which grew as well as wild‐type plants. Our results show that CCM components from Chlamydomonas can be expressed in appropriate locations in higher plant cells without compromising growth, although – consistent with modelling predictions – additional elements of the algal CCM will need to be co‐expressed to achieve enhanced productivity.

## Results

### Subcellular localization of native CCM components in Chlamydomonas

**Figure 1:**
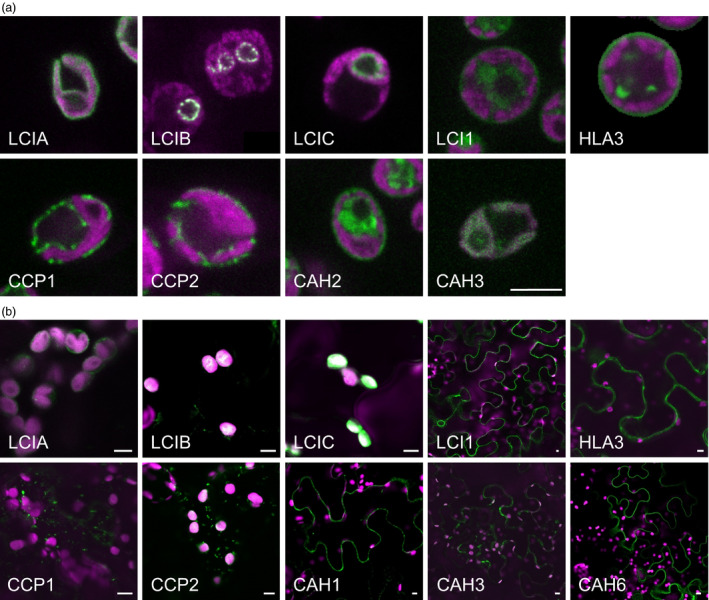
Expression of fluorescent‐tagged CCM components in Chlamydomonas and tobacco. Expression of Venus‐fused CCM components in *Chlamydomonas reinhardtii* (a). Expression in tobacco of GFP‐fused CCM components from Chlamydomonas (b). Green and purple signals are Venus or GFP fluorescence and chlorophyll autofluorescence, respectively. Overlaid images of these signals are shown: overlaps are white. Scale bar = 5 μm (all 5 μm for Chlamydomonas images). For images of separate signals see Figure S1.

The locations of CCM components in Chlamydomonas were investigated by transforming Chlamydomonas cells with constructs encoding these proteins fused to a fluorescent tag (Venus) at the C‐terminus (Figures 1a and S1a). Full‐length open reading frames were cloned from genomic DNA and constitutively expressed from the *PsaD* promoter. Most of the proteins had the subcellular locations expected from previous studies (Table 1). LCIA: Venus was confined to the chloroplast envelope; CAH3: Venus, LCIB: Venus and LCIC: Venus were in the chloroplast, with LCIC: Venus and LCIB: Venus producing the distinctive circular pattern around the pyrenoid in CO_2_‐starved cells. LCI1: Venus and HLA3: Venus were in the plasma membrane. In the absence of an available Chlamydomonas strain expressing tagged CAH1, we examined the location of the structurally related isozyme CAH2 (contiguous to CAH1 on chromosome 4, probably resulted from a gene duplication event, 91.8% identical amino acid sequences) (Fujiwara *et al*., 1990). CAH2: Venus was at the cell periphery, consistent with the expected periplasmic locations of CAH1 and CAH2 (Ynalvez *et al*., 2008). Unexpectedly, signals for CCP1: Venus and CCP2: Venus showed punctate subcellular localization, consistent with location in mitochondria. Furthermore, the location of fluorescence for CCP1: Venus and CCP2: Venus overlapped with that of a mitochondrial marker dye (Mitotracker Red CMXRos), indicating that both of these putative Ci transporters were located in mitochondria (Figure S2). There were no obvious signals for CAH6: Venus inside the Chlamydomonas cell (not shown).

### Chlamydomonas CCM proteins can be expressed in tobacco leaves

The CCM components localized in Chlamydomonas cells were selected for expression in tobacco leaves. Binary expression vectors carrying each CCM gene individually were generated by PCR amplification of cDNA and subsequent Gateway cloning (Karimi *et al*., 2002). Gene expression in tobacco was under the control of the constitutive 35S promoter and nopaline synthase (nos) terminator. Stop codons were removed to allow in‐frame C‐terminal fusion to a sequence encoding GFP. For CAH1, the N‐terminal sequence (17 aa) was replaced with a leader sequence (22 aa) from tobacco, as described by Roberts and Spalding (1995), to facilitate processing and secretion to the apoplast.

**Figure 2:**
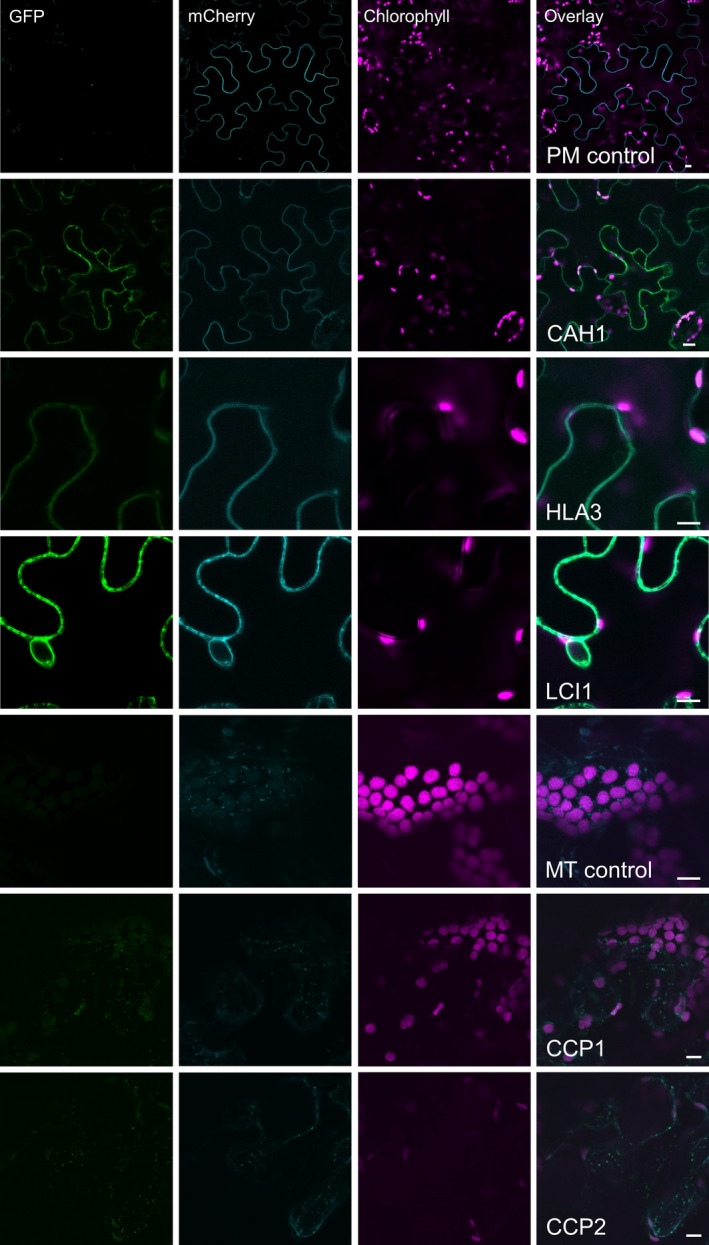
Co‐expression of GFP‐fused CCM components with a mCherry‐fused plasma membrane transporter NPSN12 or a known mitochondrial marker (the targeting sequence of yeast cytochrome oxidase IV [COX4] fused to mCherry) in tobacco. Purple, green and cyan signals are chlorophyll autofluorescence, GFP and mCherry fluorescence, respectively. Overlaid images of these signals are shown: overlaps of GFP and mCherry are pale green. PM, plasma membrane; MT, mitochondria. Scale bar = 10 μm.

The GFP‐fused CCM components were expressed transiently in tobacco leaves by agro‐infiltration. The locations of eight of the components were consistent with the demonstrated location in Chlamydomonas (Figures 1b and S1b). Fluorescent signals for LCIA: GFP were in the chloroplast envelope, and LCIB: GFP and LCIC: GFP signals were stromal. LCI1: GFP, HLA3: GFP and CAH1: GFP were at the cell periphery. The location of fluorescence for LCI1: GFP and HLA3: GFP overlapped with that of an integral plasma membrane transporter protein (NPSN12, AT1G48240) fused to mCherry (Geldner *et al*., 2009), indicating that both of these putative Ci transporters were associated with the plasma membrane (Figure 2). Although co‐expression of CAH1: GFP with NPSN12: mCherry indicated that CAH1 was located at the cell periphery, we could not resolve whether the enzyme, which is periplasmic in Chlamydomonas, was expressed discretely in the intercellular space. The fluorescence signals for CCP1: GFP and CCP2: GFP appeared predominantly in numerous discrete structures much smaller than chloroplasts. This distribution is consistent with the locations of these proteins in mitochondria, as was the case in Chlamydomonas (Figure 1a). Furthermore, fluorescence signals for CCP1: GFP and CCP2: GFP overlapped with that of a mitochondrial marker (the targeting sequence of yeast cytochrome oxidase IV [COX4] fused to mCherry [Nelson *et al*., 2007]), indicating that both of these putative Ci transporters were associated with the mitochondria (Figure 2).

The fluorescent signal for CAH3: GFP was present in both cytosol and chloroplasts when this fusion protein was expressed in tobacco. In contrast, CAH6: GFP appeared to be located exclusively in the cytosol (Figure 1b). Our own and previous data suggest that CAH3 is chloroplastic in Chlamydomonas and probably contained within the thylakoid lumen where it is suggested to play a pivotal role in the supply of CO_2_ to RuBisCO (Figure 1) (Blanco‐Rivero *et al*., 2012; Duanmu *et al*., 2009b; Karlsson *et al*., 1998; Sinetova *et al*., 2012). CAH6 has a predicted chloroplast transit peptide (TP), and there is some evidence for a chloroplast location in Chlamydomonas (Mitra *et al*., 2004). However, our CAH6: Venus protein did not have a clear intracellular location. Thus, CAH3 appears to be mistargeted when expressed in tobacco, and there is insufficient information on CAH6 to determine whether this protein is correctly localized in tobacco.

### Modification of target peptides for direction of CCM components to tobacco chloroplasts

We tested whether plasma membrane localized Ci transporters from Chlamydomonas could be retargeted to the chloroplast envelope in tobacco leaves. Screening of the plant membrane protein database (Aramemnon, http://aramemnon.botanik.uni-koeln.de/) identified a chloroplast envelope transporter belonging to the ATP‐binding cassette (ABC) superfamily: ABCI13 of Arabidopsis (AT1G65410). The predicted N‐terminal TP of ABCI13 (ABC‐TP, 60 aa) was attached to the N‐termini of HLA3 and LCI1. As controls, we used constructs encoding a Chlamydomonas protein: GFP fusion already shown to localize to the tobacco chloroplast envelope (LCIA, see above) that were modified either to remove the TP or to replace it with the ABC‐TP.

**Figure 3:**
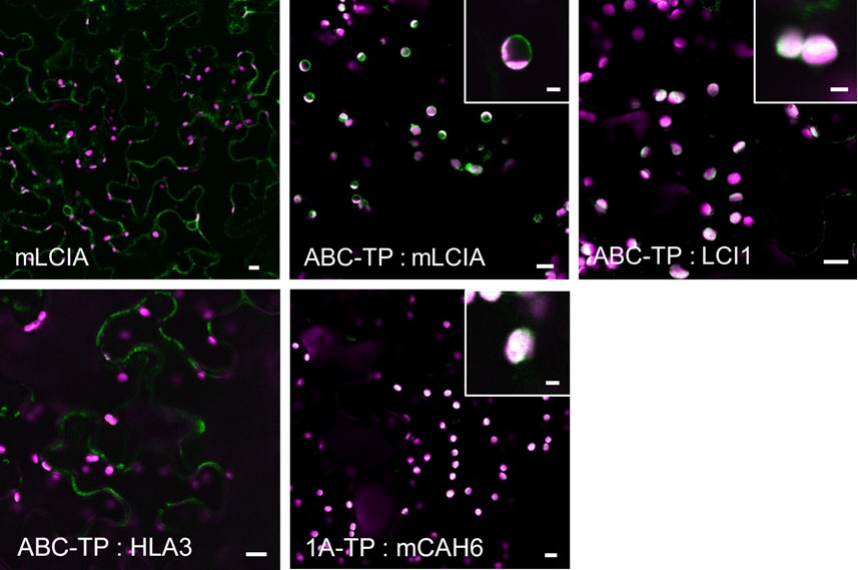
Expression of GFP‐fused CCM components carrying native Arabidopsis chloroplast transit peptides in tobacco. Green and purple signals are GFP fluorescence and chlorophyll autofluorescence, respectively. Overlaid images of these signals are shown: overlaps are white. 1A‐TP, RuBisCO small subunit RBCS1A (AT1G67090) transit peptide; ABC‐TP, ABC transporter ABCI13 (AT1G65410) transit peptide; mCAH6, mature CAH6; mLCIA, mature LCIA. Main image scale bar = 10 μm, inset image scale bar = 3 μm. For images of separate signals see Figure S3.

When expressed transiently in tobacco, LCIA: GFP lacking the predicted native LCIA‐TP (73 aa) (Miura *et al*., 2004) localized to the cytosol instead of the chloroplast (Figures 3 and S3). Chloroplast envelope targeting was recovered with a N‐terminal ABC‐TP, similarly to full‐length LCIA: GFP. This result showed that the ABC‐TP could target some transporter proteins to the chloroplast envelope. However, the addition of the ABC‐TP to LCI1: GFP or to HLA3: GFP was not sufficient to retarget these two proteins to the chloroplast envelope. The fluorescence signal for ABC‐TP: LCI1: GFP indicated that the protein was in the chloroplast stroma, whereas the signal for ABC‐TP: HLA3: GFP was in the cytosol.

Having found that CAH6: GFP did not localize to the chloroplast stroma, we replaced the native TP with the TP of the Arabidopsis RuBisCO small subunit, RBCS1A (AT1G67090). By placing the first 80 aa of RBCS1A (1A‐TP) upstream of the mature CAH6 (mCAH6) to generate 1A‐TP: mCAH6: GFP, the CAH6: GFP protein was retargeted to the chloroplast stroma (Figure 3). Thus, 1A‐TP is a suitable sequence to direct the localization of soluble Chlamydomonas proteins to the stroma.

### LCIA and HLA3 are located in the plasma membrane in Xenopus oocytes and increase Ci uptake rates

**Figure 4:**
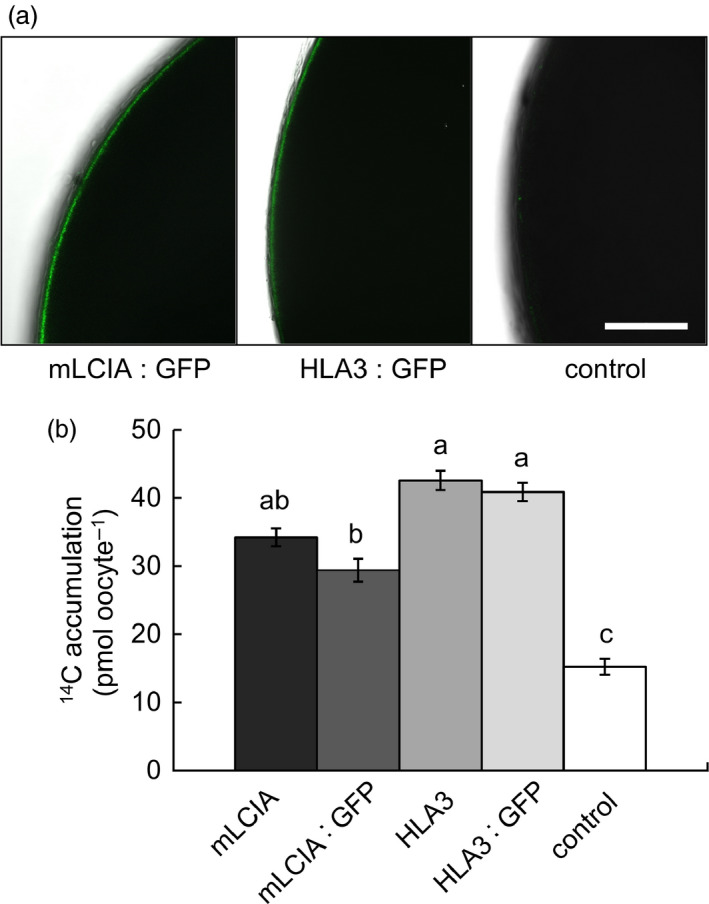
Chlamydomonas CCM components LCIA and HLA3 facilitate increased accumulation of inorganic carbon in Xenopus oocytes. Confocal images of oocytes expressing GFP fused to mature LCIA (LCIA lacking a chloroplast transit peptide, mLCIA) or HLA3 3 d after injection (a). ^14^C accumulation in oocytes expressing mLCIA or HLA3 either untagged or fused to GFP following 10‐min incubation in MBS containing 0.12 mM NaH^14^ CO_3_ (b). Values are means of measurements on 20 oocytes; bars are means ± standard error (SE). Letters above the bars indicate a difference or between values; where a, b and c indicate significant difference (*P *< 0.05) as determined by analysis of variance (ANOVA) followed by Tukey's honestly significant difference (HSD) tests.

To investigate the putative function of LCIA and HLA3 as transmembrane Ci transporters, we expressed these proteins in Xenopus oocytes, with or without N‐terminal GFP fusions. Oocytes injected with mRNA of either mature LCIA: GFP (mLCIA; lacking the N‐terminal TP) or HLA3: GFP displayed a fluorescent signal on the cell surface after 3 d, indicating protein expression and incorporation into the plasma membrane (Figure 4a). mRNA‐injected cells were assayed for Ci uptake using H^14^CO_3_^‐^ as a tracer. Oocytes transformed with mLCIA: GFP or HLA3: GFP accumulated 2.0‐ and 2.7‐fold more ^14^C than water‐injected controls, respectively (Figure 4b). The presence of a GFP‐tag had no adverse effect on H^14^CO_3_^‐^ uptake.

### LCIA and HLA3 express in appropriate locations in Arabidopsis leaf cells following stable transformation

**Figure 5:**
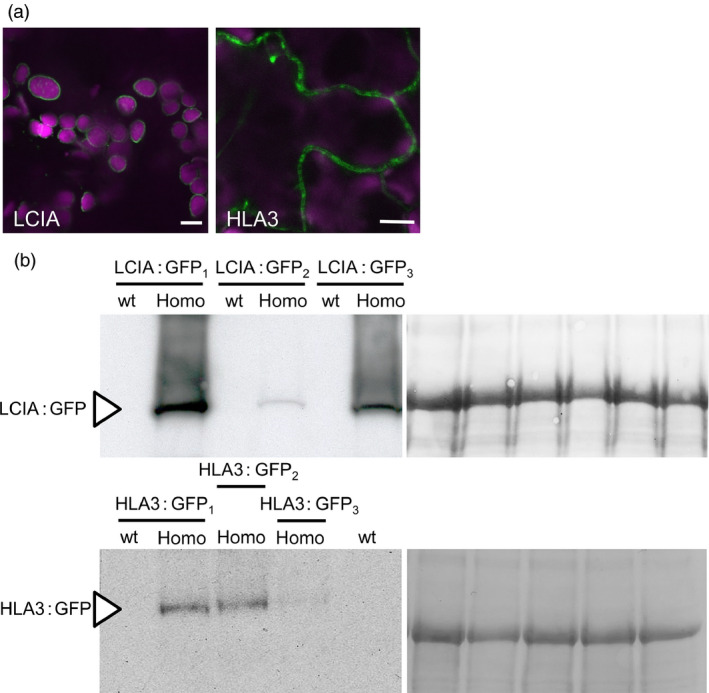
Stable expression of LCIA: GFP and HLA3: GFP in Arabidopsis. Representative confocal images of LCIA and HLA3 fused to GFP (a). Green and purple signals are GFP fluorescence and chlorophyll autofluorescence, respectively. Overlaid images of these signals are shown: overlaps are white. Scale bar = 10 μm. For images of separate signals see Figure S4. Immunoblots of rosette extracts (10 μg protein) from LCIA: GFP‐ and HLA3: GFP‐expressing lines probed with an antibody against GFP (b). LCIA: GFP is present in three separate homozygous T3 insertion lines (LCIA: GFP_1‐3_), but not in segregating wild‐type lines. HLA3: GFP is visible in HLA3: GFP_1‐3_ but not in the segregating wild‐type for HLA3: GFP_1_ or a wild‐type equivalent for HLA3: GFP_2_ and HLA3: GFP2_3_. LCIA: GFP and HLA3: GFP have approximate masses of 54 and 170 kDa, respectively (arrow). Ponceau stains of each blot (right) show the band attributable to the RuBisCO large subunit (RbcL, 55 kD) as a loading control.

Following transformation by floral dip, three *Arabidopsis thaliana* (ecotype Columbia; Col‐0) homozygous T3 lines with stable expression of either LCIA: GFP or HLA3: GFP were selected for further study. Both fusion proteins resulted in fluorescent signals in the same subcellular locations as in tobacco leaves (Figures 5a and S4). Leaf proteins were separated on SDS‐PAGE and probed after blotting with a commercial antibody raised against GFP. Polypeptides corresponding to 54 kDa for LCIA: GFP and 170 kDa for HLA3: GFP were resolved (Figure 5b). These masses are consistent with those expected for GFP (27 kDa) fusions of LCIA after TP cleavage (27.5 kDa) and HLA3 (147 kDa) (Yamano *et al*., 2015).

### Transgenic Arabidopsis have normal growth and photosynthetic characteristics

**Figure 6:**
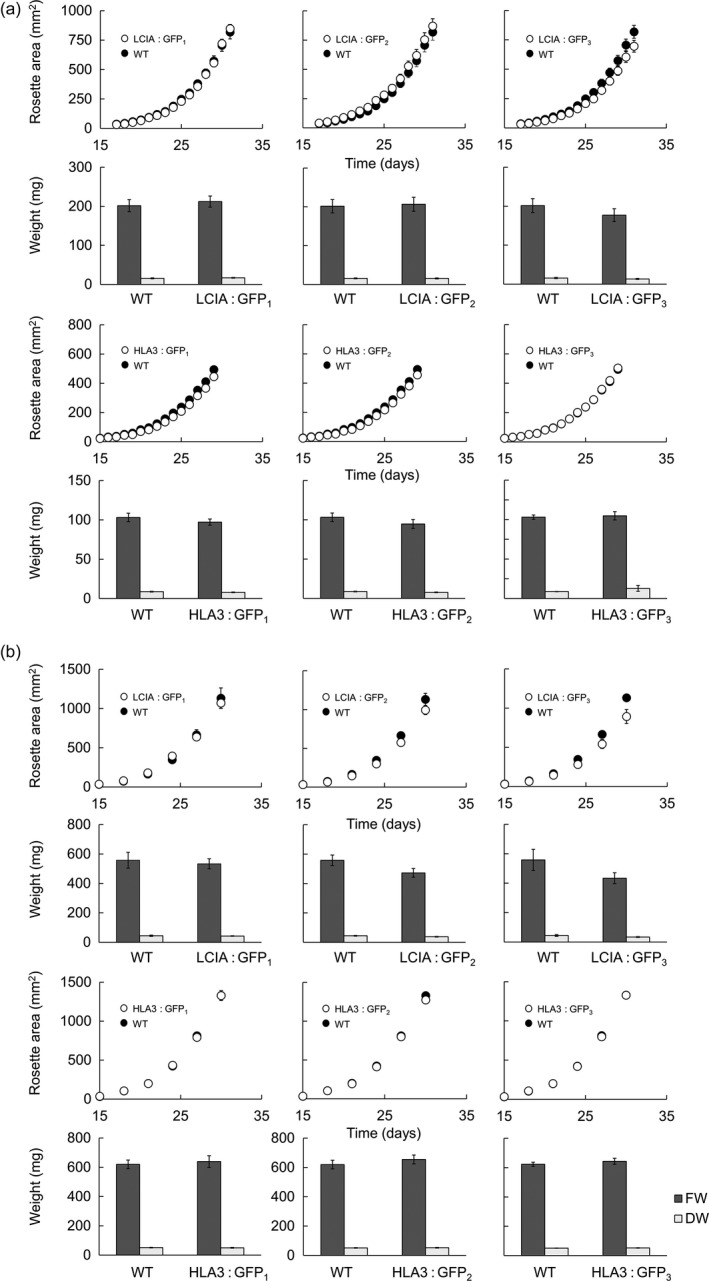
Growth of phenotypes in different environmental conditions of transgenic Arabidopsis plants expressing LCIA or HLA3. Plants were grown under ambient CO_2_ (*ca*. 400 μmol/mol) and 100 μmol photons/m^2^/s (a) or low CO_2_ (250 μmol/mol) and 350 μmol photons/m^2^/s (b). Growth rates (1st and 3rd row) and fresh weight (FW) and dry weight (DW) (2nd and 4th row) are shown for LCIA and HLA3, respectively. HLA3 transgenic lines had a lower FW and DW compared to LCIA when grown under ambient CO_2_, as plants were harvested slightly earlier (at 29 days vs 31 days). All plants grown under low CO_2_ were harvested at 30 days. Values are the means ± SE of measurements made on 24 rosettes.

Wild‐type plants and LCIA: GFP‐ or HLA3: GFP‐expressing lines were grown together under ambient CO_2_ (*ca*. 400 μmol/mol) and a light intensity of 100 μmol photons/m^2^/s (Figure 6a). Growth rate was compared by measuring rosette expansion, fresh weight and dry weight. No differences were observed between wild‐type and transgenic lines. Furthermore, there was no difference in leaf chlorophyll content (Figure S5). To investigate whether the presence of algal Ci transporters affected growth rate under conditions in which chloroplastic CO_2_ concentration is expected to be a major limitation on photosynthesis, plants were grown under low CO_2_ (250 μmol/mol) and high light (350 μmol photons/m^2^/s) (Figure 6b). All genotypes had higher growth rates and lower specific leaf areas under these conditions (Figure S6), but again, there were no significant differences in growth between wild‐type and transgenic lines.

**Figure 7:**
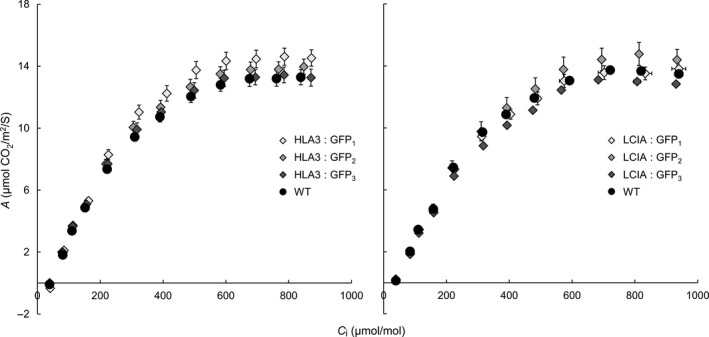
Photosynthetic responses of transgenic plants. Photosynthetic rates were determined as a function of increasing substomatal CO_2_ concentrations (*A*/*C*_i_) at saturating light levels (1500 μmol photons/m^2^/s). Each curve represents the means ± SE of values from four leaves, each on a different plant.

We checked whether expression of LCIA: GFP or HLA3: GFP affected the steady‐state rate of photosynthetic CO_2_ assimilation at ambient CO_2_ (*A*_n_) (Table 2). We also measured the relationship between the rate of photosynthesis and the CO_2_ concentration in the leaf substomatal cavity (A/*C*_i_, Figure 7) and used this to infer other photosynthetic parameters including the diffusion of CO_2_ from substomatal cavity to chloroplast (mesophyll conductance, *g*_m_) (Griffiths and Helliker, 2013) and the maximum RuBisCO carboxylation rate (*V*_c,max_). There were no significant differences in any of the parameters between transgenic lines expressing LCIA: GFP or HLA3: GFP and wild‐type plants.

**Table 2:** Photosynthetic parameters determined from gas exchange analysis of LCIA or HLA3 transgenic plants. Values are the mean ± SE of measurements made on four leaves, each from a different plant (as shown in Figure 7)

	Wild type	HLA3: GFP_1_	HLA3: GFP_2_	HLA3: GFP_3_	wild type	LCIA: GFP_1_	LCIA: GFP_2_	LCIA: GFP_3_
*A*_n_ (μmol CO_2_/m^2^/s)	9.4 ± 0.6	11 ± 0.9	10 ± 0.8	9.9 ± 0.8	9.7 ± 0.2	9.4 ± 0.5	9.7 ± 1.2	8.8 ± 0.2
*g*_s_ (mol CO_2_/m^2^/s)	0.21 ± 0.04	0.29 ± 0.03	0.21 ± 0.03	0.25 ± 0.04	0.26 ± 0.03	0.24 ± 0.04	0.25 ± 0.05	0.24 ± 0.01
*g*_m_ (mol CO_2_/m^2^/s)	0.047 ± 0.003	0.048 ± 0.005	0.047 ± 0.003	0.044 ± 0.005	0.04 ± 0.001	0.038 ± 0.002	0.04 ± 0.004	0.034 ± 0.001
*V*_c,max_ (μmol CO_2_/m^2^/s)	30 ± 3.2	30.4 ± 2.4	29.3 ± 1.8	27.3 ± 2.2	28.7 ± 1.1	29.2 ± 2.8	31.4 ± 2.3	27 ± 0.7
*J*_max_ (μmol e^‐^/m^2^/s)	64.5 ± 4.8	71.6 ± 5.6	67 ± 4.7	64.3 ± 5.2	64.5 ± 1.9	65.1 ± 4.6	69.5 ± 6.6	61.5 ± 1.1
Γ (μmol CO_2_/mol)	39.5 ± 2.5	44 ± 2.6	37.9 ± 1.4	36.1 ± 2.6	33.2 ± 0.6	31.5 ± 2.4	32.9 ± 0.9	29.1 ± 1
Initial slope (*A*_n_/*C*_i)_	0.044 ± 0.002	0.046 ± 0.004	0.044 ± 0.003	0.043 ± 0.004	0.038 ± 0.001	0.035 ± 0.002	0.038 ± 0.005	0.036 ± 0.001

*A*_n_, net photosynthesis at ambient CO_2_; *g*_s_, stomatal conductance to CO_2_; *g*_m_, mesophyll conductance to CO_2_; *V*_c,max_, maximum velocity of RuBisCO carboxylation; *J*_max_, maximum capacity of electron transport; Γ, CO_2_ compensation point.

ANOVA revealed that there were no statistically significant differences between samples (*P *< 0.05).

## Discussion

The introduction of a microbial eukaryotic CCM into crop plants will require robust methods to ensure that the proteins of interest perform their intended function in the foreign host system. To that effect, we developed a pipeline to (i) determine the native subcellular location in Chlamydomonas, (ii) test the targeting efficiency of constructs by transient expression in tobacco and (iii) provide a platform for physiological characterization of transgenic lines by stably transforming Arabidopsis. Through this approach, we showed that eight CCM components from Chlamydomonas can be successfully transferred into appropriate locations in leaves of higher plants. Confirmation of subcellular localization of CCM components is an important step in systematic introduction of a fully operational CCM into higher plants (Pengelly *et al*., 2014). For example, successful expression and assembly of components of the cyanobacterial carboxysome microcompartment in tobacco chloroplasts will help to guide future attempts to assemble the cyanobacterial CCM into higher plants (Lin *et al*., 2014a,b). In contrast, transfer of ictB, a putative cyanobacterial CCM component, into Arabidopsis and tobacco plants has resulted in enhanced biomass accumulation (Lieman‐Hurwitz *et al*., 2003; Simkin *et al*., 2015), but lack of information on ictB protein structure, function and cellular localization in transgenic plants limits our understanding of this growth response (Simkin *et al*., 2015).

The need to establish robustly the native location of CCM components, prior to incorporation into higher plants, is further supported by our work on two putative chloroplast membrane transporters. The suggestion that CCP1 and CCP2 are in the chloroplast envelope was based on their enrichment in chloroplast preparations and in preparations of envelope membranes (Mason *et al*., 1990; Ramazanov *et al*., 1993). The possibility of a mitochondrial location was not explicitly examined in these studies. Here, our robust co‐expression approach revealed that tagged CCP1/2 proteins were associated with mitochondria rather than the chloroplast envelope in both Chlamydomonas and tobacco (Figures 1 and 2; Figures S1 and S2). This location is also consistent with the high degree of similarity between CCP1/2 and the mitochondrial carrier protein superfamily (Pollock *et al*., 2004; Pfam 00153; KOG0758). The expression of CCP1 and CCP2 is strongly induced by low CO_2,_ both genes are under the control of the CCM ‘master switch’ *Cia5/Ccm1*, and reducing CCP1/2 expression (through an RNAi approach) resulted in slower growth rates at low CO_2_. However, cells with reduced CCP1/2 proteins did not lose CCM or photosynthetic capacity at low CO_2_ (Pollock *et al*., 2004). Taken together, these results suggest that transport across mitochondrial membranes plays an important role in coordinating the CCM with growth‐related metabolism. In Chlamydomonas, mitochondria are known to relocate from a central position within the cup of the chloroplast to a peripheral position close to the plasma membrane when cells grown in high CO_2_ are exposed to limiting CO_2_ (Geraghty and Spalding, 1996). It has been suggested that mitochondrial relocation may be important either for energization of plasma membrane Ci transporters (to date, plasma membrane HLA3 is the only ATP‐dependent Ci transporter candidate) or for scavenging glycolate produced in photorespiration during acclimation to limiting CO_2_ (Spalding, 2009). A better understanding of the role of CCP1/2 in Chlamydomonas will inform future engineering strategies for higher plants.

The location of the carbonic anhydrases CAH3 and CAH6 in tobacco also differed from predicted results (Figure 2). The partial cytosolic localization of CAH3 is likely an effect of the failure of the higher plant import machinery to recognize the dual signalling peptide of this protein. In Chlamydomonas (Figure 1), results were consistent with location of the protein in the thylakoid lumen (Duanmu *et al*., 2009b; Sinetova *et al*., 2012). In particular, the presence of fluorescence in the centre of the pyrenoid is consistent with the suggestion that CAH3 is concentrated in the transpyrenoidal thylakoid tubules following low CO_2_‐induced phosphorylation (Figure 1) (Blanco‐Rivero *et al*., 2012). In contrast, there is no clear evidence of a role for CAH6 in the Chlamydomonas CCM, and very little is known about its function. CAH6 represents the only apparent stromal carbonic anhydrase activity and has been adopted into the current CCM model as a speculative mechanism for Ci uptake and/or recovery of CO_2_ escaping from the pyrenoid (Wang *et al*., 2015; Yamano *et al*., 2010). Evidence for chloroplast location in Chlamydomonas is based on a predicted stroma‐signalling peptide and on immunogold labelling (Mitra *et al*., 2004), but we did not observe an intracellular location for a CAH6: Venus protein.

Our attempts to relocate CCM components in tobacco cells by modifying targeting sequences were successful for some components. CAH6 could be redirected from the cytosol to the chloroplast stroma in tobacco by removal of the predicted TP and fusion to the TP of the Arabidopsis RuBisCO small subunit 1A, 1A‐TP (Figure 3). The plasma membrane protein LCI1 could be relocated to the chloroplast by fusion with the TP of an Arabidopsis chloroplast envelope transporter, and the same TP could substitute for the native TP of the Chlamydomonas chloroplast envelope protein LCIA. However, this TP did not redirect the plasma membrane protein HLA3 to the chloroplast envelope. Moving larger transmembrane proteins like HLA3 to the chloroplast may require further modifications to remove potential competing signal motifs and to ensure correct orientation in the case of directional channels.

The putative Ci transporters HLA3 and LCIA localized to the plasma membrane and chloroplast envelope, respectively, in Chlamydomonas, tobacco and Arabidopsis (Figures 1 and 5). Recent work has confirmed these locations in Chlamydomonas (Yamano *et al*., 2015). We showed that both proteins can facilitate uptake of Ci into Xenopus oocytes, consistent with previous demonstrations that LCIA can facilitate Ci movement across membranes (Mariscal *et al*., 2006) and that HLA3 overexpression contributes to increased Ci uptake in Chlamydomonas (Gao *et al*., 2015; Yamano *et al*., 2015). Further kinetic analyses will be required to determine the mode of action of these proteins (i.e. active *vs*. passive transport). This information will be crucial for modelling approaches and rational engineering strategies. For example, rational approaches for engineering the cyanobacterial CCM into higher plants are based on a good understanding of the catalytic characteristics of cyanobacterial Ci transporters BicA and SbtA (Du *et al*., 2014; McGrath and Long, 2014; Price *et al*., 2011, 2013).

The addition of LCIA or HLA3 did not confer a growth advantage to Arabidopsis plants (Figures 6 and 7, Table 2). This is not entirely unexpected, as biophysical CCMs, in both algae and cyanobacteria, require additional features to function, including a microcompartment containing RuBisCO and additional components to reduce Ci leakage (Badger *et al*., 1998; Price *et al*., 2013; Yamano *et al*., 2010). Furthermore, it is likely that native carbonic anhydrase activity, particularly in the stroma, would hinder Ci accumulation. Biophysical CCMs rely on the capacity to accumulate bicarbonate; thus, specific localization and control of carbonic anhydrase activity appear to play an important role in functionality. For example, ectopic expression of carbonic anhydrase within the cytoplasm of cyanobacterial cells leads to a debilitating leakage of Ci due to rapid equilibration between bicarbonate and CO_2_ in the cytosol (Price and Badger, 1989). Predictive models indicate that the removal of native stromal carbonic anhydrases is a key target for introducing biophysical CCMs into higher plants (McGrath and Long, 2014; Price *et al*., 2013). Our results do demonstrate that higher plant cells can express Ci transporters from Chlamydomonas in appropriate locations, without deleterious effect. Both LCIA and HLA3 showed appropriate transmembrane integration and increased Ci uptake when expressed in Xenopus oocytes (Figures 1, 4 and 5). Furthermore, we were able to detect both proteins in transgenic Arabidopsis leaves (Figure 5). Together, these data suggest that LCIA and HLA3 were active, but that activity was not sufficient to drive up CO_2_ levels around RuBisCO and hence improve the rate of CO_2_ assimilation.

Recent work has shown that expression and function of HLA3 and LCIA may be closely coordinated in Chlamydomonas (Yamano *et al*., 2015). Enhanced Ci uptake was observed when both proteins were overexpressed in wild‐type Chlamydomonas at high CO_2_. However, negligible changes in photosynthesis and Ci uptake were observed when either HLA3 or LCIA was overexpressed, indicating that, in the absence of other CCM components or a microcompartment such as a pyrenoid, these proteins are not able to enhance chloroplastic CO_2_ concentrations (Gao *et al*., 2015; Yamano *et al*., 2015). These data suggest that subsequent engineering strategies in higher plants should focus on co‐expression of HLA3 and LCIA. Additional modifications will probably be required to establish a functional biophysical CCM in higher plants, including the removal of native carbonic anhydrases, which would otherwise short circuit active uptake mechanisms and eliminate any stromal pool of bicarbonate (Price *et al*., 2013). The next challenge will be to stack key CCM components, and develop a strategy to retarget stromal RuBisCO to a chloroplast microcompartment, a predicted requirement for an algal‐type CCM (Badger *et al*., 1998).

## Experimental procedures

### Plant material and growth conditions

Mutant and transgenic plants of Arabidopsis (*Arabidopsis thaliana*) were in the Col‐0 wild‐type background. Arabidopsis seeds were sown on compost, stratified for 3 days at 4 °C and grown at 20 °C, under ambient CO_2_ (*ca*. 400 μmol/mol), at 70% relative humidity and under 100 μmol photons/m^2^/s in 12‐h light/12‐h dark cycles, unless otherwise stated.

For analyses of transgenic lines, homozygous insertion or wild‐type out‐segregant lines (T3) were compared. Where wild‐type out‐segregants were not available, homozygous insertion lines were compared with Col‐0 plants from seed stocks of the same age generated under similar conditions. Tobacco plants (*Nicotiana benthamiana* L.) were cultivated under glass house conditions (minimum 20 °C, natural light supplemented to give at least 12‐h light). Venus‐tagged proteins were expressed in wild‐type Chlamydomonas strain cMJ030 (CC‐4533)(Zhang *et al*., 2014). Cells were maintained in constant low light (~10 μmol photons/m^2^/s) at RT on 1.5% (w/v) agar plates containing Tris–acetate–phosphate (TAP) (Kropat *et al*., 2011). For imaging, cells were grown in liquid TAP media to a concentration of 10^6^ cells/mL, pelleted by centrifugation (1000 *g*, 4 min), resuspended in Tris–phosphate (T‐P) minimal media (Kropat *et al*., 2011) and grown for 24 h in ambient CO_2_ before imaging.

### Cloning and expression of CCM components in Chlamydomonas

The open reading frames (ORFs) of Chlamydomonas genes were expressed in frame with Venus from the *PsaD* promoter using the pLM005 vector. ORFs were amplified from genomic DNA using Phusion Hotstart II polymerase (Thermo Fisher Scientific, www.thermofisher.com) with the respective oligos in Table S1. HpaI‐cut pLM005 vector and PCR products were gel purified and assembled by Gibson assembly (Gibson *et al*., 2009). Due to the large gene length of *HLA3,* it was cloned in two fragments then assembled in the pLM005 vector by Gibson assembly. The pLM005 vector contains the AphVIII gene for paromomycin resistance in Chlamydomonas and ampicillin resistance for bacterial selection. All construct junctions were verified by Sanger sequencing. Constructs were transformed into Chlamydomonas by electroporation as in Zhang *et al*. (2014). Briefly, 250 μL of 2 × 10^8^ cells/mL was transformed with 14.5 ng/kbp of EcoRV‐cut plasmid at 16 °C. Cells were spread on 86 mL TAP agar plates containing paromomycin (20 μg/mL) and kept in low light (~10 μmol photons/m^2^/s) until colonies were ~2–3 mm in diameter. Plates were screened for fluorescent colonies using a Typhoon TRIO fluorescence scanner (GE Healthcare, www.gelifesciences.com) with excitation/emission wavelengths 532 nm/520–555 nm for Venus and 633 nm/630–670 nm for chlorophyll autofluorescence.

### Cloning and expression of CCM components in tobacco and Arabidopsis

Genes were cloned from cDNA derived from Chlamydomonas (strain CC‐4886, Chlamydomonas Resource Center). Primers were designed from sequences available on Phytozome v10.2 (*Chlamydomonas reinhardtii* v5.5 [Augustus u11.6], http://phytozome.jgi.doe.gov/pz/portal.html#!info?alias=Org_Creinhardtii) (see Table S1 for oligo details). Gene sequences for *LCIA* and *HLA3* were codon‐optimized for expression in higher plants and synthesized *de novo* (DNA2.0, CA, USA) (Figure S7), then cloned into Gateway entry vectors (pCR^®^8/GW/TOPO^®^TA Cloning^®^ Kit) using Platinum^®^ Taq DNA Polymerase High Fidelity according to the manufacturer's instructions (Invitrogen^™^ Life Technologies, www.lifetechnologies.com) and subsequently cloned into the destination binary vectors pK7FWG2,0 (Karimi *et al*., 2002) or pGWB5 (Nakagawa *et al*., 2009). Gibson assembly was used to generate transit peptide gene fusions. Binary vectors were transformed into *Agrobacterium tumefaciens* (AGL1) for transient gene expression in tobacco leaves (Schöb *et al*., 1997) or stable insertion in Arabidopsis plants by floral dipping (Clough and Bent, 1998). Co‐expression studies were performed with the WAVE131 vector of the ‘wave’ marker set (Geldner *et al*., 2009) and the mt‐rb vector (Nelson *et al*., 2007) for plasma membrane and mitochondria localization, respectively.

### DNA extraction, PCR and protein analysis

For screening transgenic Arabidopsis lines, genomic DNA was extracted from mature, nonflowering rosettes of T1 plants as described in Li and Chory (Li and Chory, 1998). PCRs were performed as in McCormick and Kruger (2015). Where possible, the location of gene inserts was confirmed by TAIL PCR as described by Liu *et al*. (1995). Homozygous insertion lines were identified in the T2 generation either by PCR or by seedling segregation ratios on kanamycin‐containing Murashige and Skoog (MS) medium (0.5x) plates.

Relative levels of LCIA: GFP and HLA3: GFP proteins in leaves were confirmed by immunoblot. Approximately 10 μg protein from whole 28‐d‐old rosettes (100 mg fresh weight) was fractionated by SDS‐PAGE on a 10% (w/v) acrylamide: bisacrylamide (40:1) gel transferred to PVDF membrane, probed with mouse anti‐GFP IgG_2a_ at 1:1,000 dilution (Santa Cruz, http://www.scbt.com/) and visualized using an HRP‐conjugated goat anti‐mouse IgG_2a_ at 1:50 000 dilution. HRP activity was detected using Supersignal Ultra (Pierce, www.piercenet.com) according to the manufacturer's instructions.

### Oocyte expression and bicarbonate uptake assays

Synthesized gene sequences for mature *LCIA* (mLCIA) or *HLA3* lacking a stop codon were cloned into the expression vector Vivid Colors^™^ pcDNA^™^ 6.2/EmGFP (Invitrogen^™^ Life Technologies) to generate mLCIA‐ or HLA3‐GFP fusions. For LCIA, the N‐terminal transit sequence peptide (73 aa) was removed and replaced with the sequence ‘GACATG’ to add a Kozak sequence and new start codon. For HLA3, ‘GAC’ was added immediately upstream of the start codon. To generate equivalent vectors lacking a fluorescent tag, Gibson assembly was used to add a stop codon to mLCIA or HLA3 and remove the GFP sequence (720 bp). Plasmids were linearized by AvrII or StuI (Roche, www.roche.co.uk) and capped mRNA was synthesized using the mMESSAGE mMACHINE^®^ T7 Transcription Kit (Ambion^®^; Life Technologies) according to the manufacturer's instructions. Mature LCIA or HLA3 mRNA was expressed in oocytes as described by Feng *et al*. (2013). Xenopus oocytes were injected with 50 nL of mRNA (1 μg/μL) or diethyl pyrocarbonate (DEPC)‐treated water as a control. Bicarbonate uptake assays and confocal imaging were performed 3 d after injection in a protocol adapted from Mariscal *et al*. (2006). Oocytes were incubated in fresh MBS media (88 mm NaCl, 1 mm KCl, 2.4 mm NaHCO_3_, 0.71 mm CaCl_2_, 0.82 mm MgSO_4_ and 15 mm HEPES, pH 7.4), containing 0.12 mm NaHCO_3_ (1.85 GBq/mol NaH^14^CO_3_). After 10 min, the oocytes were washed three times with ice‐cold MBS and lysed in 200 μL SDS (10% [w/v]), and the radioactivity retained in individual oocytes was measured.

### Chlorophyll quantification

Chlorophyll was extracted from powdered leaf discs in ice‐cold 80% (v/v) acetone and 10 mm Tris–HCl, and concentration was measured according to Porra *et al*. (1989).

### Measurement of photosynthetic parameters

Gas exchange rates were determined using a LI‐6400 portable infrared gas analyser (LI‐COR Biosciences, http://www.licor.com/) on either the sixth or seventh leaf of 35‐ to 45–d‐old mature, nonflowering rosettes grown in large pots to generate leaf area sufficient for gas exchange measurements (Flexas *et al*., 2007). The response of net photosynthetic CO_2_ assimilation (*A*) to substomatal CO_2_ concentration (*C*_i_) was measured by varying the external CO_2_ concentration from 0 to 1000 μmol/mol under a constant photosynthetic active radiation of 1500 μmol photons/m^2^/s (provided by a red–blue light source attached to the leaf chamber). Gas exchange data were corrected for CO_2_ diffusion as in Bellasio *et al*. (2015). Leaf temperature and chamber relative humidity were maintained at 21 °C and 70%, respectively. To calculate maximum carboxylation rate (*V*_c,max_), maximum electron transport flow (*J*_max_) and mesophyll conductance (*g*_m_), the A/Ci data were fitted to the C_3_ photosynthesis model (Farquhar *et al*., 1980) with modifications to include estimations for *g*_m_ as described by Ethier and Livingston (2004).

### Confocal laser scanning microscopy

A Leica TCS SP2 laser scanning confocal microscope (Leica Microsystems) with a water immersion objective lens (HCX IRAPO 25.0x0.95) was used for imaging leaves and oocytes. Excitation/emission wavelengths were 488 nm/500–530 nm for GFP, 543 nm/590–620 nm for mCherry and 488 nm/680–750 nm for chlorophyll autofluorescence. Images were acquired using Leica LAS AF software (http://www.leica-microsystems.com/). Prior to imaging Venus‐tagged proteins in Chlamydomonas, 15 μL of cells were added to a well of a 96‐well optical plate (Brooks Life Science Systems, http://www.brooks.com) and covered with 150 μL of 1.5% low melting point agarose containing T‐P (~35 °C). For mitochondria staining, CCP1: Venus‐ and CCP2: Venus‐expressing lines were grown in liquid T‐P media with paromomycin (2 μg/mL) to a concentration of 2–4 × 10^6^ cells/mL. Cultures were incubated with MitoTracker Red CMXRos (Thermo Fisher Scientific) to a final concentration of 1 μm for 10 min, then spotted on a polylysine‐coated slide for imaging. Cells were imaged using a custom adapted confocal microscope (Leica DMI6000) with settings at 514 nm/532–555 nm for Venus, 561 nm/573–637 nm for staining by Mitotracker Red CMXRos and 561 nm/665–705 nm for chlorophyll autofluorescence. Images were analysed using Fiji software (http://fiji.sc/Fiji).

### Statistical analysis

Variations in response between genotypes were assessed by either analysis of variance (ANOVA) or Student's *t*‐tests followed by Tukey's honest significant difference (HSD) post hoc test (SPSS Statistics 18, http://www.ibm.com/). Differences for which *P *< 0.05 are considered significant.

## Supporting information


**Figure S1** Expression of fluorescent‐tagged CCM components in Chlamydomonas and tobacco (from Figure 1).
**Figure S2** Co‐expression of Venus‐fused CCM components with Mitotracker Red CMXRos in Chlamydomonas.
**Figure S3** Expression of GFP‐fused CCM components carrying native Arabidopsis chloroplast transit peptides in tobacco (from Figure 3).
**Figure S4** Stable expression of LCIA: GFP and HLA3: GFP in Arabidopsis (from Figure 5a).
**Figure S5** Chlorophyll content of transgenic Arabidopsis plants expressing LCIA or HLA3.
**Figure S6** Specific leaf area (area/DW) of transgenic LCIA or HLA3 Arabidopsis plants.
**Figure S7** DNA sequences of codon‐optimized LCIA and HLA3.
**Table S1** Sequences of synthetic oligonucleotides used in this study.
