# Supplementary material for: Introducing an algal carbon‐concentrating mechanism into higher plants: location and incorporation of key components
Source: Plant Biotechnol J. 2015 Nov 5;14(5):1302–15. doi: 10.1111/pbi.12497 (PMC5102585; doi:10.1111/pbi.12497)
Supplement: Supplementary file 1 — Figure S1 Expression of fluorescent‐tagged CCM components in Chlamydomonas and tobacco (from Figure 1). Figure S2 Co‐expression of Venus‐fused CCM components with Mitotracker Red CMXRos in Chlamydomonas. Figure S3 Expression of GFP‐fused CCM components carrying native Arabidopsis chloroplast transit peptides in tobacco (from Figure 3). Figure S4 Stable expression of LCIA: GFP and HLA3: GFP in Arabidopsis (from Figure 5a). Figure S5 Chlorophyll content of transgenic Arabidopsis plants expressing LCIA or HLA3. Figure S6 Specific leaf area (area/DW) of transgenic LCIA or HLA3 Arabidopsis plants. Figure S7 DNA sequences of codon‐optimized LCIA and HLA3. Table S1 Sequences of synthetic oligonucleotides used in this study. [file PBI-14-1302-s001.pdf]

(a)

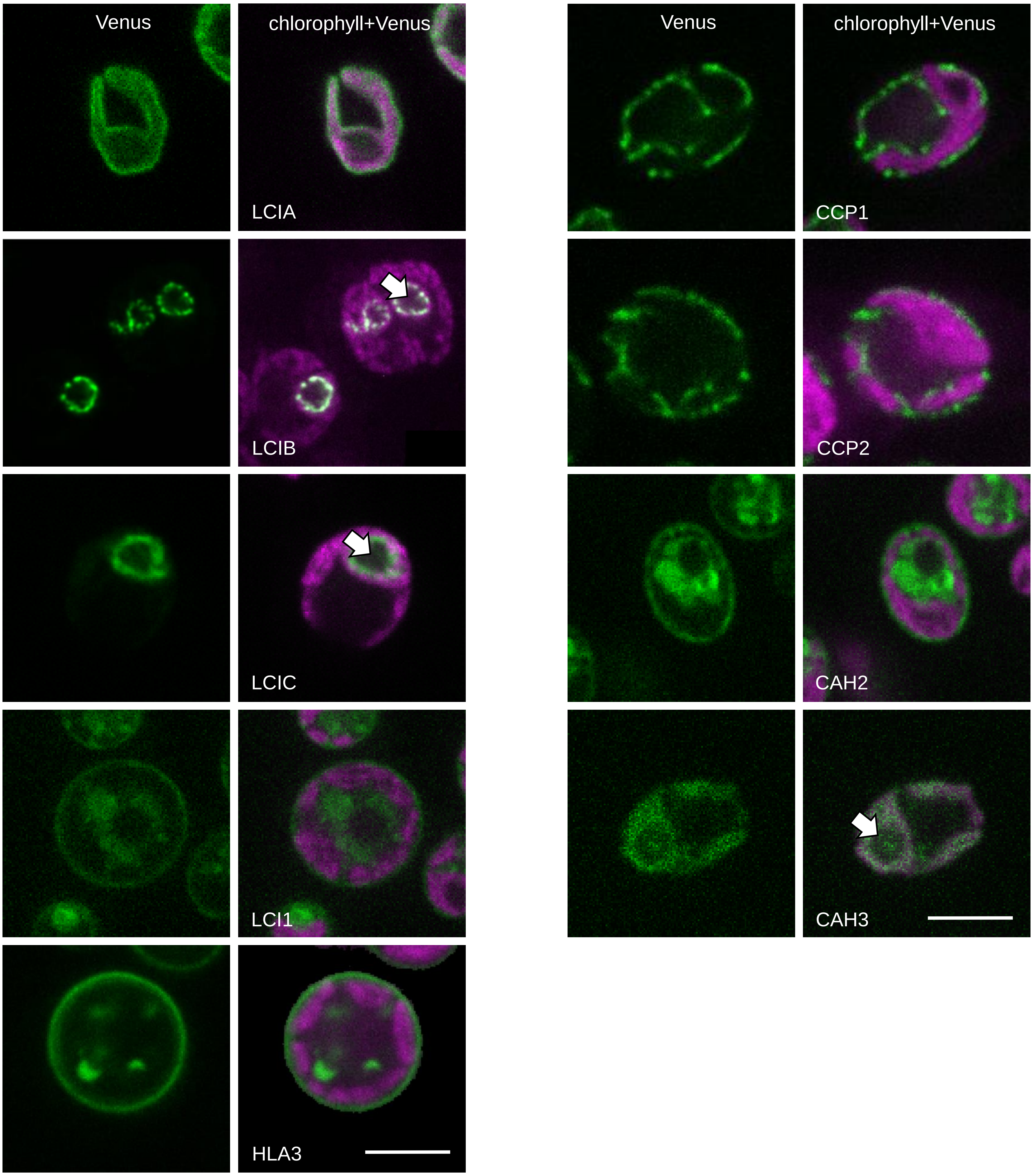

Venus
chlorophyll+Venus
LCIA
LCIB
LCIC
LCI1
HLA3
Venus
chlorophyll+Venus
CCP1
CCP2
CAH2
CAH3

(b)

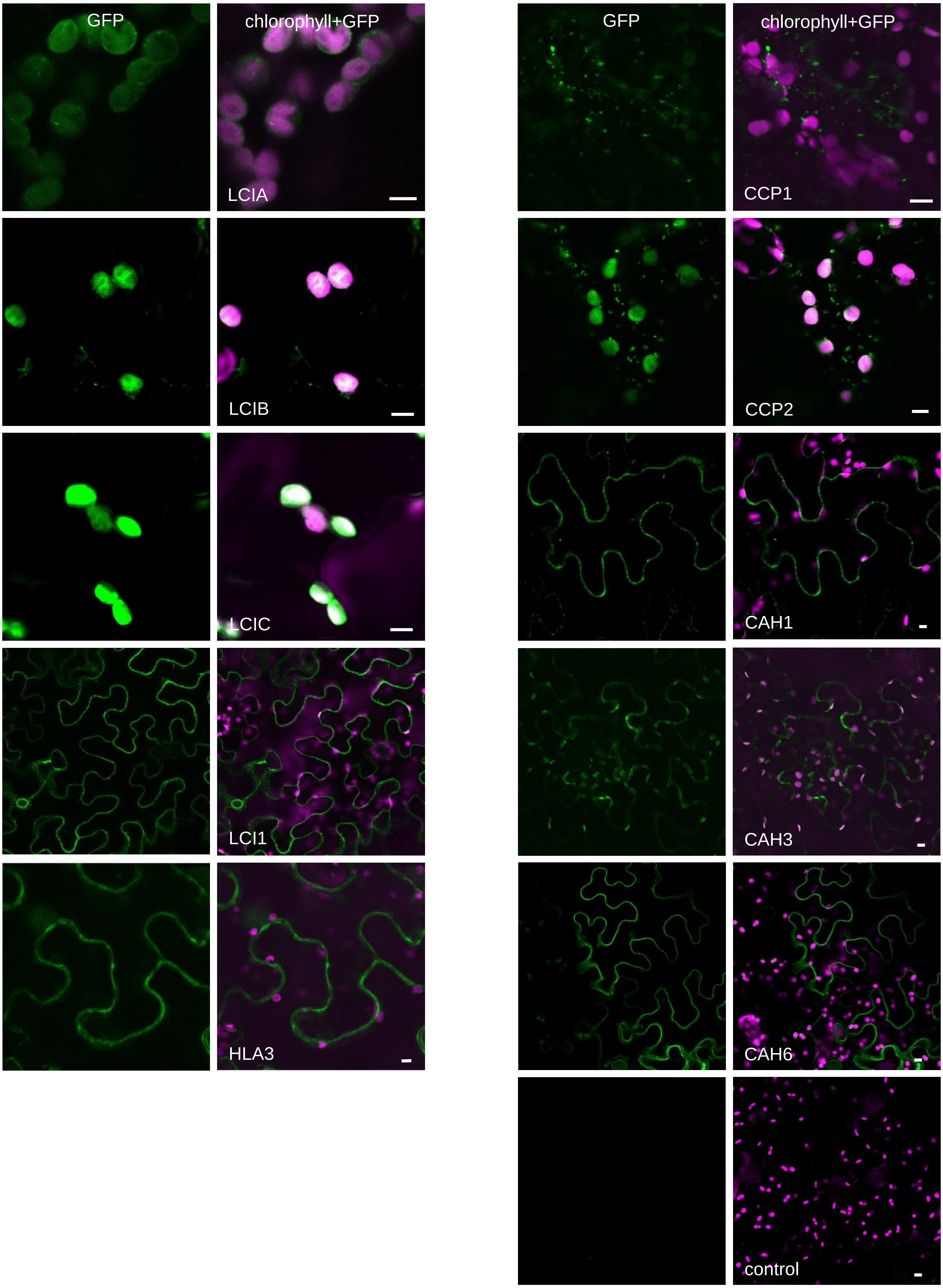

Figure S1. Expression of fluorescent-tagged CCM components in Chlamydomonas and tobacco (from Fig. 1). Expression of Venus-fused CCM-components in *Chlamydomonas reinhardtii* (a). Expression in tobacco of GFP-fused CCM components from Chlamydomonas (b). Green and purple signals are Venus or GFP fluorescence and chlorophyll autofluorescence, respectively. Overlaid images of these signals are shown: overlaps are white. Arrowhead shows the label-free area is the algal pyrenoid, a micro-compartment composed primarily of RuBisCO. A non-transformed control is included. Scale bar = 5 µm (all 5 µm for Chlamydomonas images).

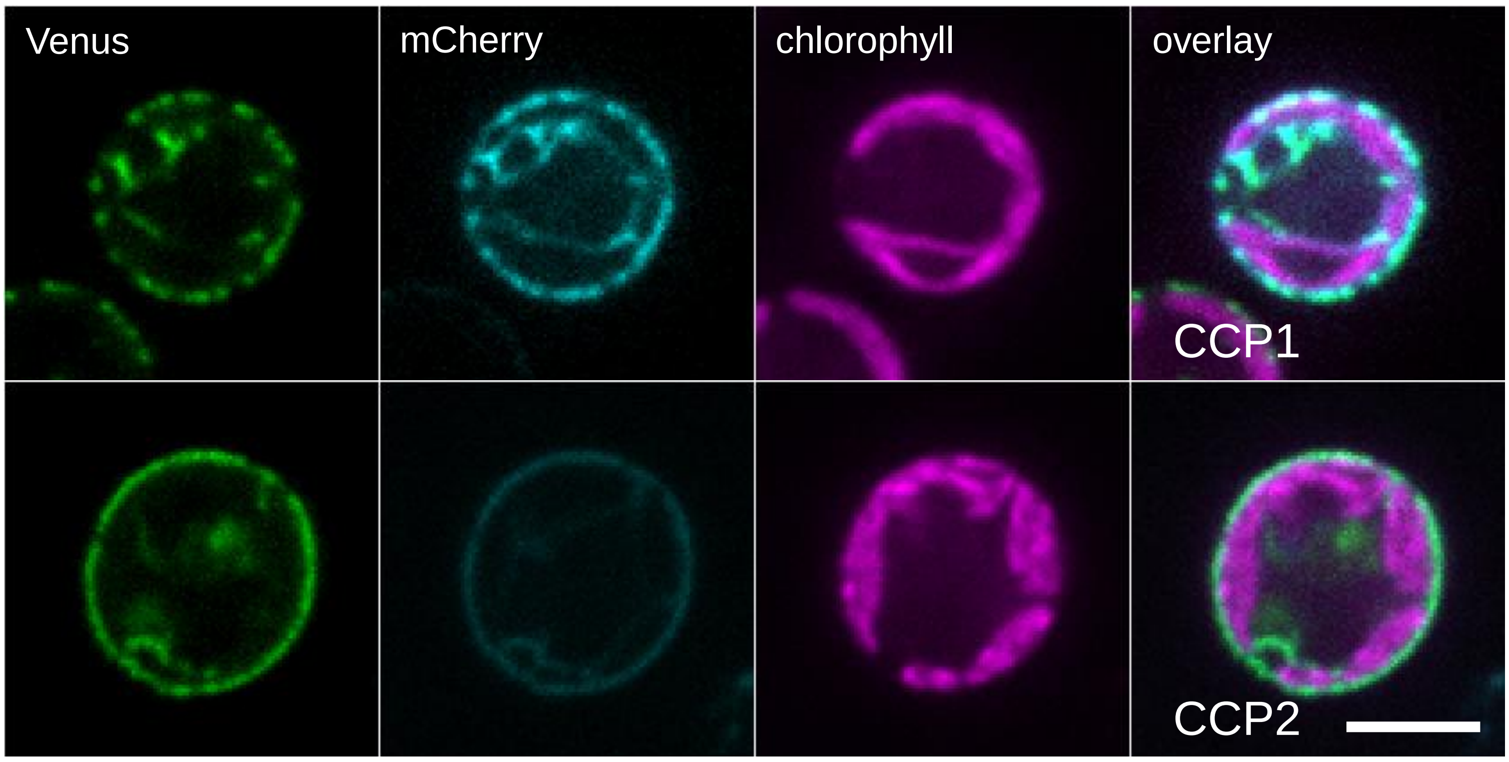

Figure S2. Co-expression of Venus-fused CCM components with Mitotracker® Red CMXRos in Chlamydomonas. Purple, green and cyan signals are chlorophyll autofluorescence, Venus and Mitotracker fluorescence, respectively. Overlaid images of these signals are shown: overlaps of Venus and Mitotracker are pale green. Scale bar = 5 µm.

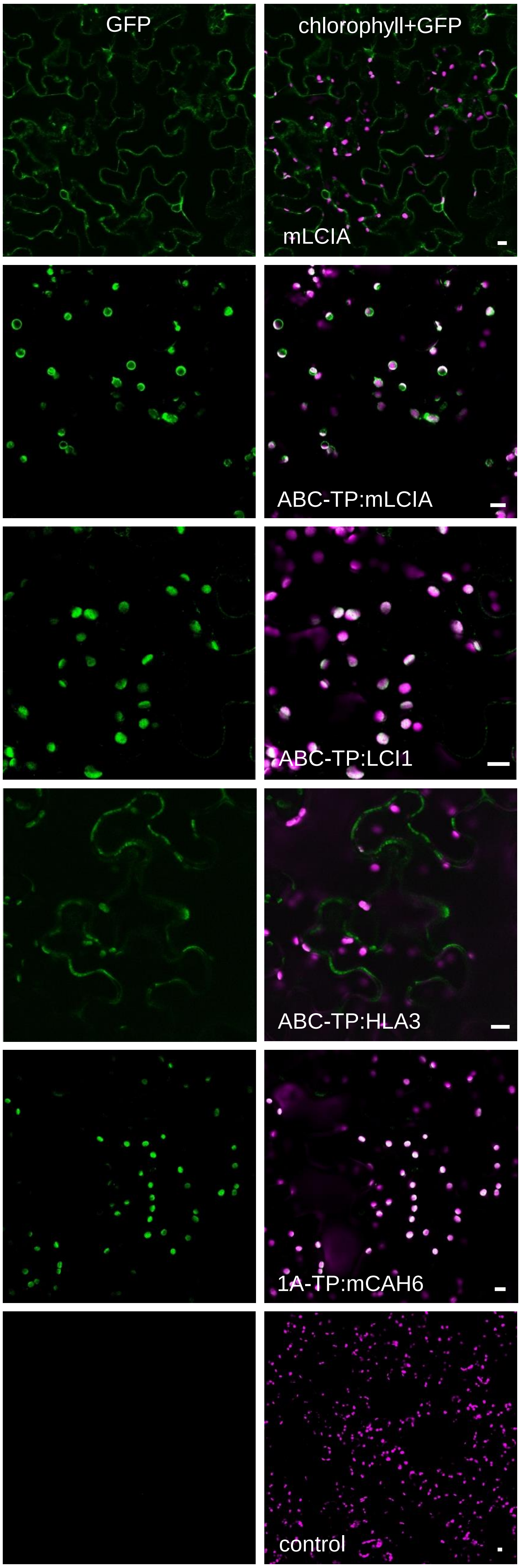

Figure S3. Expression of GFP-fused CCM components carrying native Arabidopsis chloroplast transit peptides in tobacco (from Fig. 3). Green and purple signals are GFP fluorescence and chlorophyll autofluorescence, respectively. Overlaid images of these signals are shown: overlaps are white. A non-transformed control is included. Scale bar = 10 µm.

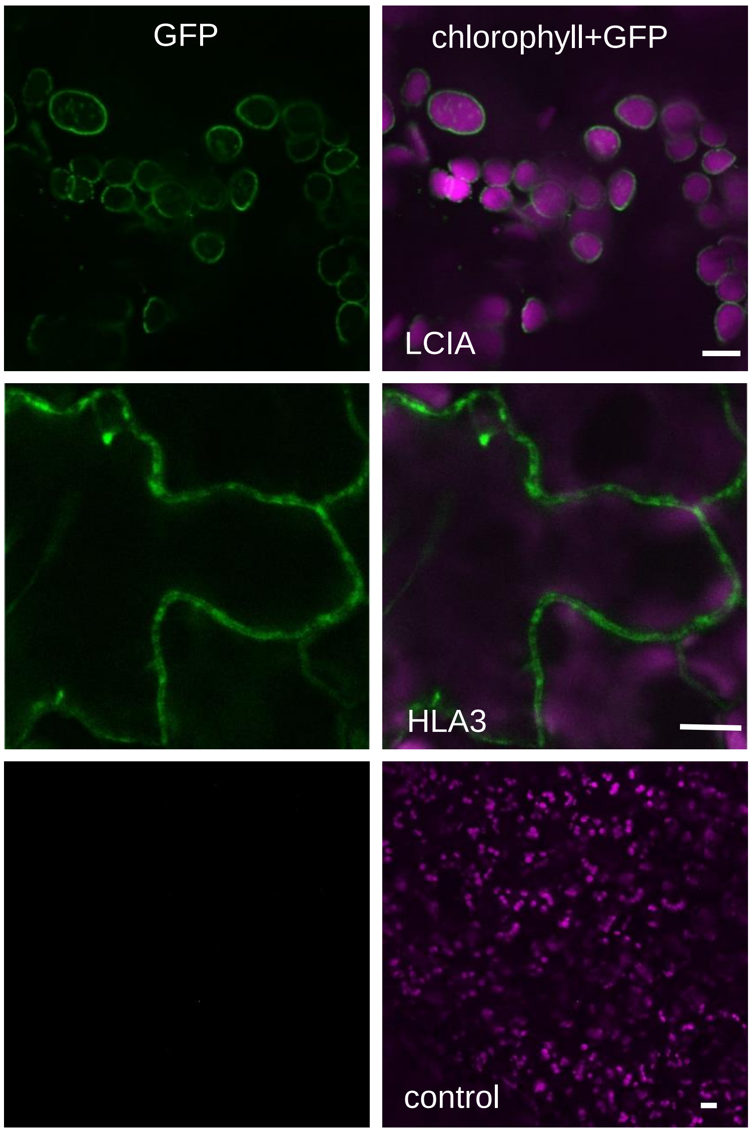

Figure S4. Stable expression of LCIA:GFP and HLA3:GFP in Arabidopsis (from Fig. 5a). Green and purple signals are GFP fluorescence and chlorophyll autofluorescence, respectively. Overlaid images of these signals are shown: overlaps are white. A non-transformed control is included. Scale bar = 10 µm.

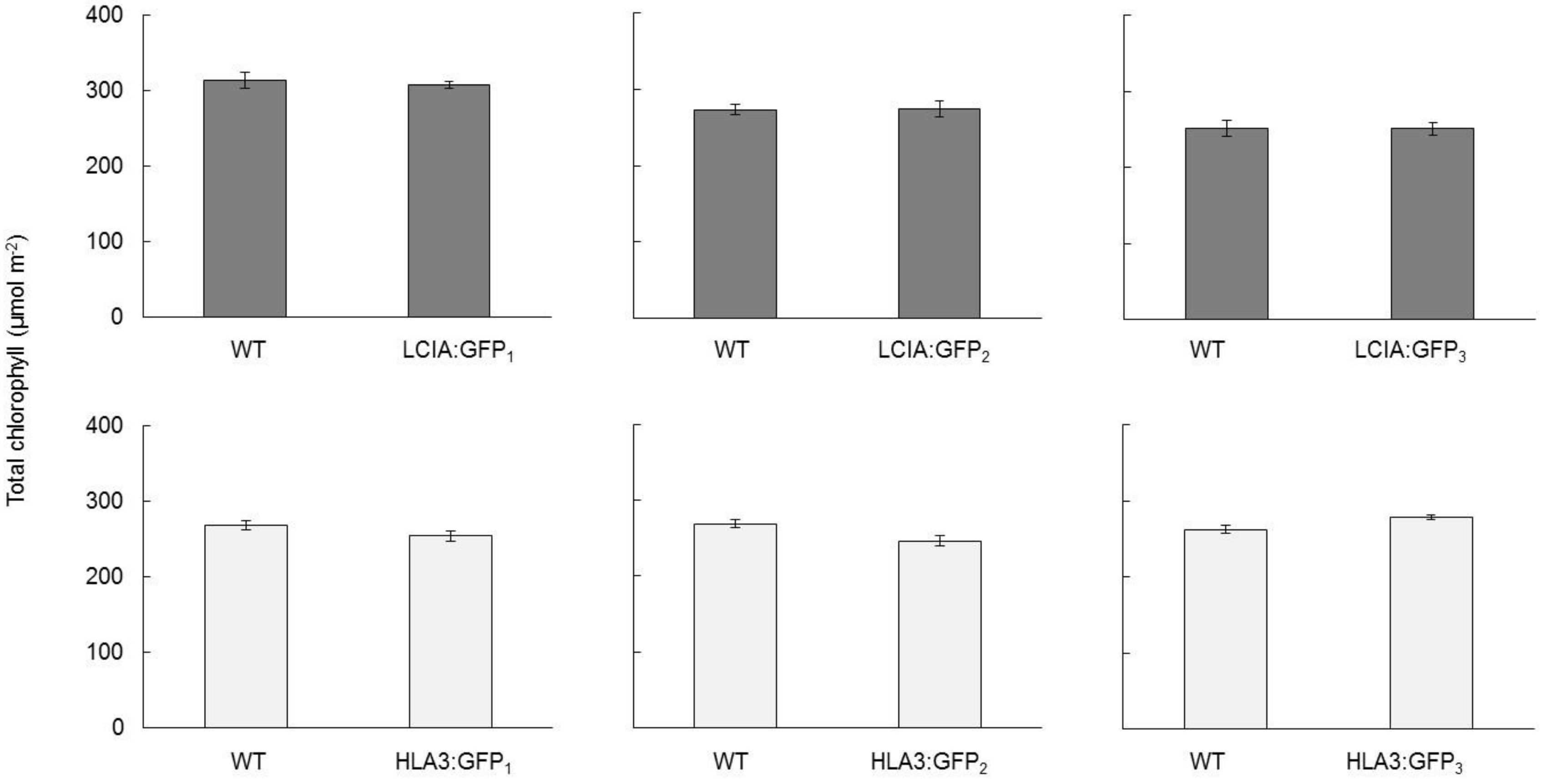

Figure S5. Chlorophyll content of transgenic Arabidopsis plants expressing LCIA or HLA3. Chlorophyll content measurements are shown for 8 separate plants, bars are means ± SE. ANOVA revealed that there were no statistically significant differences between samples (P< 0.05).

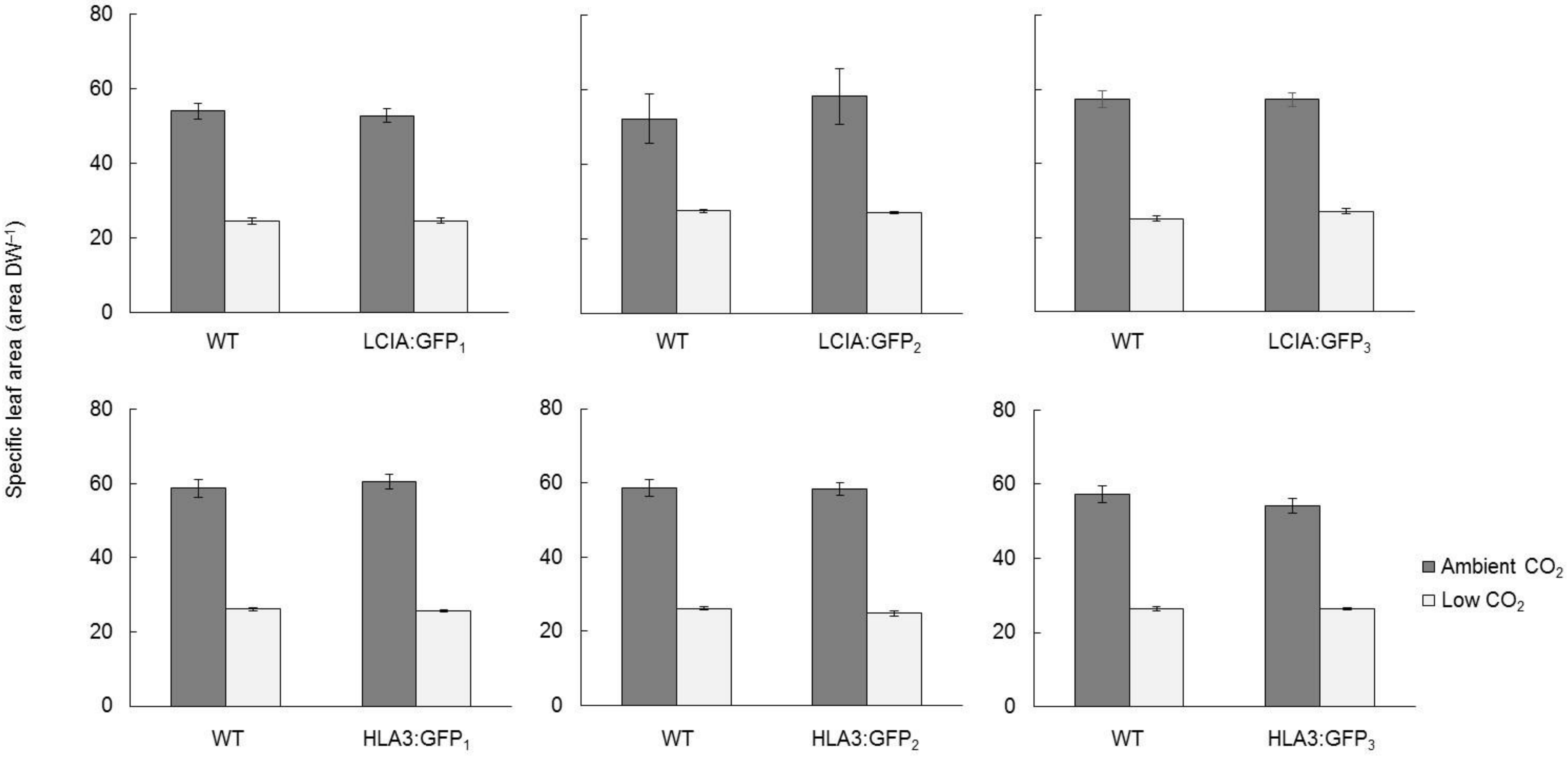

Figure S6. Specific leaf area (area/DW) of transgenic LCIA or HLA3 Arabidopsis plants. Values are the means ± SE of measurements made on 24 rosettes. Growth conditions refer to those outlined in Fig. 6.

Codon optimised LCIA (1011 bp)

ATGCAGACAACTATGACAAGACCTTGTTTAGCCCAGCCAGTGTTACGATCCAGAGTTCTTAGATCGCCAATGC
GGGTTGTTGCTGCTAGTGCTCCGACAGCTGTGACAACTGTCGTCACCTCAAATGGAAATGGGAACGGGCACTT
TCAGGCGGCTACGACTCCTGTGCCACCAACGCCAGCTCCTGTAGCTGTGTCTGCGCCTGTCAGGGCTGTTAGT
GTTCTGACACCCCCTCAAGTTTATGAAAACGCAATTAACGTTGGAGCCTATAAAGCTGGACTTACTCCGCTGGC
AACATTCGTACAAGGTATTCAAGCCGGTGCATACATCGCATTTGGCGCTTTCTTAGCTATAAGCGTCGGCGGC
AATATCCCGGGAGTTGCTGCTGCAAACCCTGGACTAGCAAAGTTGCTATTTGCTCTCGTGTTTCCTGTCGGACT
TTCGATGGTGACTAATTGTGGTGCAGAACTTTTCACCGGTAACACCATGATGTTGACATGCGCCTTAATCGAG
AAAAAGGCCACTTGGGGGCAACTCCTTAAGAACTGGTCTGTGTCTTACTTTGGTAACTTCGTTGGTAGTATCG
CTATGGTAGCGGCAGTTGTTGCCACCGGTTGCCTCACCACGAATACTCTACCAGTTCAAATGGCGACACTTAA
GGCGAATTTGGGATTCACTGAGGTTTTAAGTCGTTCAATCCTCTGTAATTGGTTGGTATGTTGTGCAGTTTGGT
CAGCCTCCGCAGCGACGAGCCTTCCGGGAAGGATTCTTGCTCTTTGGCCCTGCATCACTGCCTTCGTGGCAAT
AGGATTGGAACATTCCGTGGCTAATATGTTTGTAATTCCACTCGGGATGATGCTAGGAGCGGAGGTGACTTG
GAGCCAGTTTTTCTTTAACAACTTGATTCCCGTTACGCTGGGCAATACCATAGCTGGTGTCTTGATGATGGCAA
TAGCATATTCTATTTCATTCGGTTCTTTGGGTAAATCTGCTAAACCTGCTACAGCATGA

Codon optimised HLA3 (3978 bp)

ATGGGAGAGGGGAAGGTGGCCGAGAGAGGTCATGATCCTACTGCAGGCTTCTTCAACAAGTTTGCTTTCGGG
TGGATGTACAAATGGATTGGTGCTGCTAGGCGAGGTGAAGAACTTAATGCCGAAGAAATGGGTATGCCACCA
GAAAACATGGCACACGAGGCTTATGATAAGTTTGCGGCCCATTGGGCTGCGGAGCAGAAACTTAAAGACCAG
GATGGGCAGAAGCCGTCATTGGTAAGGGCTCTTAGAAAATCATTTGGTCTATTCTACATGTTGGGAGGTCTTT
GCAAATGCGGATGGTCAACATTCGTGATTCTTGGTGCTTTTTACTTTGTGCGTTCACTCTTGTCGCATGTCAACT
ATGTAGATGCTCCTACGAAAAAGTGGACAACGGCTGAAACTTTGTATGGCCAAACAACCGCTGGATGGCTCTT
AATGATGGGTTTCACAATAGATGCTTGGCTTTTGGGACTTTGTCTCCAAAGAATGGGTTACATATGCATGACG
GTTGGTATCCGTGCGAGAGCTGCTCTTGTCCAAGCGGTTACTCATAAAGCTTTCCGACTTAATACGGTTAGAG
CTGATCAATCAGCCGCGATTGTCAACTTCGTGGCTTCCGATATCCAGAAAATCTACGATGGGGCGTTAGAGTT
TCATTATCTTTGGACCGCCCCTTTTGAAGCCGCAGCAATCCTTGCCCTCTTGGGATACTTGACACAAGACTCCAT
GCTCCCAGGATTGGGGGTCATTTTGCTAGTGTTGCCTATGCAGTATTACTTCGGTTATAAGATTGTACAAATCA
AGCTTCAAAACGCTAAGCATGTTGCTCTCCGGTCAGCAATTATGCAGGAAGTGCTTCCTGCTATCAAGCTTGTT
AAGTACTACGCATGGGAACAGTTCTTCGAGAACCAAATCTCAAAAGTACGTAGGGAGGAAATAAGGTTGAAC
TTCTGGAATTGCGTCATGAAGGTCATTAACGTTGCATGTGTATTTTGTGTTCCGCCTATGACAGCCTTTGTGAT
CTTTACCACTTATGAGTTTCAACGCGCTAGATTAGTATCTTCTGTGGCGTTTACTACACTCAGCTTATTCAATAT
CCTGCGCTTTCCGTTGGTTGTTTTGCCAAAGGCTCTGAGAGCTGTTTCTGAAGCTAACGCTAGCTTGCAAAGAT
TGGAAGCGTATTTGTTGGAAGAGGTTCCATCTGGAACAGCAGCAGTAAAGACTCCTAAGAATGCGCCACCGG
GAGCGGTCATTGAAAACGGGGTGTTTCATCATCCCTCTAACCCAAATTGGCATCTCCACGTACCAAAGTTTGA
GGTAAAGCCAGGTCAGGTGGTCGCTGTTGTTGGTCGGATTGCCGCTGGTAAGAGCTCGTTGGTACAAGCTAT
ATTGGGTAATATGGTTAAGGAACACGGTTCCTTCAATGTTGGTGGCCGGATCAGCTACGTACCTCAGAATCCT
TGGCTCCAAAACCTTTCTTTGCGAGACAACGTGCTTTTTGGTGAACAATTTGACGAGAATAAGTACACGGATG
TCATTGAATCTTGTGCATTGACTCTGGACCTTCAGATACTTTCCAACGGGGATCAGAGTAAAGCTGGTATTAGA
GGAGTGAATTTCTCCGGTGGGCAAAGACAAAGGGTCAACCTTGCTCGTTGTGCCTACGCAGACGCCGACCTT
GTCCTGTTGGACAACGCGCTGTCCGCGGTGGATCATCATACGGCGCATCACATTTTTGATAAATGTATCAAAG
GATTATTCAGTGATAAAGCCGTAGTGCTCGTTACTCACCAGATTGAGTTCATGCCTAGGTGTGATAACGTTGCT
ATCATGGATGAGGGCCGATGTCTCTACTTCGGTAAGTGGAACGAAGAGGCTCAACATCTTCTGGGAAAATTG
CTCCCTATCACACATCTCCTTCACGCCGCAGGCTCGCAGGAAGCTCCTCCAGCACCGAAGAAAAAGGCAGAAG
ATAAGGCAGGGCCACAAAAGTCACAGAGTTTACAGTTAACACTTGCCCCAACCTCTATAGGAAAGCCCACCGA
AAAACCAAAAGATGTTCAAAAGCTTACGGCCTACCAGGCTGCACTTATCTATACCTGGTATGGAAATTTGTTCT

TGGTGGGAGTTTGTTTCTTCTTCTTCCTGGCTGCTCAATGTTCCCGCCAGATCTCAGACTTTTGGGTGAGGTGG
TGGGTTAATGATGAATACAAGAAGTTTCCTGTCAAGGGAGAACAAGACTCGGCTGCAACTACCTTTTACTGCT
TGATATATCTTCTACTAGTTGGTCTGTTTTACATTTTCATGATCTTTAGGGGTGCGACCTTTCTCTGGTGGGTAT
TGAAAAGCTCCGAAACAATTCGTCGTAAGGCACTGCATAACGTCCTAAATGCTCCAATGGGGTTCTTCCTTGTC
ACACCAGTTGGAGATCTGTTGCTTAACTTTACAAAAGATCAAGACATAATGGATGAAAATCTTCCGGACGCCG
TGCACTTTATGGGAATCTATGGCTTGATATTGTTAGCCACCACTATAACTGTAAGTGTGACTATTAACTTCTTCG
CTGCTTTTACAGGTGCGTTGATAATCATGACGCTCATCATGTTATCAATCTATCTGCCAGCTGCTACAGCACTTA
AGAAAGCAAGAGCAGTGTCAGGTGGGATGCTAGTGGGACTTGTTGCTGAAGTCTTGGAGGGGCTCGGTGTG
GTGCAAGCATTCAATAAGCAAGAGTATTTCATTGAAGAGGCTGCTAGACGTACAAACATCACTAATAGTGCTG
TTTTCAACGCAGAGGCTTTGAACCTCTGGTTAGCATTTTGGTGTGACTTCATTGGTGCTTGCTTGGTGGGCGTC
GTTTCTGCGTTTGCAGTTGGGATGGCAAAGGATTTAGGTGGCGCTACTGTTGGTCTGGCATTTTCTAATATCAT
TCAAATGCTCGTGTTCTATACTTGGGTGGTTAGATTCATTTCAGAGTCTATCTCGCTATTCAATAGCGTTGAGG
GAATGGCTTATTTGGCAGATTATGTGCCACACGATGGAGTGTTTTATGACCAGCGTCAAAAAGATGGAGTCGC
TAAGCAAATAGTTTTACCGGATGGTAATATCGTCCCAGCGGCTTCCAAGGTTCAAGTTGTTGTGGATGACGCA
GCGTTGGCTCGATGGCCTGCTACAGGAAATATCAGATTTGAAGATGTGTGGATGCAATATCGTTTAGATGCCC
CTTGGGCGCTTAAAGGCGTAACTTTCAAAATCAATGACGGTGAGAAGGTGGGTGCAGTGGGAAGAACAGGA
TCTGGTAAAAGTACAACGCTTTTAGCTCTGTACAGGATGTTCGAACTCGGAAAAGGGAGAATCTTAGTCGACG
GCGTGGATATAGCTACATTGTCTTTGAAGAGACTCCGTACCGGGCTCAGTATTATCCCCCAAGAGCCAGTTAT
GTTCACAGGAACTGTTCGGTCTAATCTGGATCCCTTTGGAGAGTTCAAGGATGACGCCATACTTTGGGAAGTG
CTCAAGAAAGTTGGATTAGAGGACCAGGCTCAGCACGCCGGTGGATTGGACGGACAAGTCGACGGAACTGG
TGGCAAAGCTTGGTCGCTCGGACAAATGCAATTGGTATGCCTTGCTAGAGCAGCACTCAGAGCCGTGCCCATT
TTGTGTCTTGATGAGGCCACTGCAGCGATGGATCCTCATACTGAAGCAATTGTTCAACAGACGATAAAGAAAG
TATTCGATGACCGGACCACTATCACTATCGCACATCGGTTAGATACGATCATAGAAAGCGACAAGATTATCGT
TATGGAGCAAGGCTCTCTTATGGAATATGAATCGCCTAGTAAGCTGCTGGCTAACCGAGATTCGATGTTCTCC
AAACTCGTTGATAAAACTGGACCTGCAGCTGCGGCAGCTCTACGTAAGATGGCTGAAGATTTTTGGAGTACGC
GTTCAGCGCAAGGCCGTAATCAATGA

Figure S7. DNA sequences of codon-optimised LCIA and HLA3.

Table S1. Sequences of synthetic oligonucleotides used in this study.

Gene name	Phytozome 10.2 Cr v5.5 (Augustus u11.6)	Gene length (bp)	Forward primer	Reverse primer	Internal forward primer	Internal reverse primer
**Full-length ORFs**						
LCIA	Cre06.g309000	2001	GCTACTCACAACAAGCCCAGTTATGCAGACCAC TATGACTCGCCCTTG	GAGCCACCCAGATCTCCGTTCGCGGTGGCG GGCTTGG		
LCIB	Cre10.g452800	2172	GCTACTCACAACAAGCCCAGTTATGTTCGCTCTG TCTTCGC	GAGCCACCCAGATCTCCGTTGTTCTTGGGG GCCTCGAAG		
LCIC	Cre06.g307500	2161	GCTACTCACAACAAGCCCAGTTATGGCCCTTGC TCAGAAGATGAACGTG	GAGCCACCCAGATCTCCGTTGTTCATGTTGG CGAGCTCGGGC		
LCI1	Cre03.g162800	1616	GCTACTCACAACAAGCCCAGTTATGTCGGGCTT GAACAAGTTCATC	GAGCCACCCAGATCTCCGTTCACCTTGGTG GCGCCGTC		
HLA3*	Cre02.g097800	6068	GCTACTCACAACAAGCCCAGTTATGGGCGAGGG CAAGGTGGCC	GAGCCACCCAGATCTCCGTTCTGGTTGCGG CCCTGCGCG	ACTTCTCCGGTGGC CAGCGC	CAGCACCAGGTCGG CGTCGG
CCP1	Cre04.g223300	1891	GCTACTCACAACAAGCCCAGTTATGTCATCCGA CGCTATGACTATCAACGA	GAGCCACCCAGATCTCCGTTCTGCGCGGCG GTGGGCG		
CCP2	Cre04.g222750	1801	GCTACTCACAACAAGCCCAGTTATGGCATCCGA CGCTATGACTATCAACG	GAGCCACCCAGATCTCCGTTCTGAGCGGTG GCGGGGCC		
CAH2	Cre04.g223050	3309	GCTACTCACAACAAGCCCAGTTATGGCGCGTAC TGGCGCTCTAC	GAGCCACCCAGATCTCCGTTCTCGTAGCGG GCCAGCTTGATG		
CAH3	Cre09.g415700	1594	GCTACTCACAACAAGCCCAGTTATGCGCTCAGC CGTTCTAC	GAGCCACCCAGATCTCCGTTCAGCTCGTATT CGACCAGG		
CAH6	Cre12.g485050	1226	GCTACTCACAACAAGCCCAGTTATGGGATGCGG TGCCAGCGTG	GAGCCACCCAGATCTCCGTTCTCGGCGCTG CTGCCGCC		
**cDNA**						
LCIA	Cre06.g309000	1008	ATGCAGACCACTATGACTCGCC	CGCGGTGGCGGGCTTGGC		
LCIB	Cre10.g452800	1344	ATGTTCGCTCTGTCTTCGCG	GTTCTTGGGGGCCTCGAAG		
LCIC	Cre06.g307500	1329	ATGGCCCTTGCTCAGAAGATG	GTTCATGTTGGCGAGCTCG		
LCI1	Cre03.g162800	576	ATGTCGGGCTTGAACAAGTTCATCTATGTGG	CACCTTGGTGGCGCCGTCAG		
HLA3	Cre02.g097800	3975	ATGGGCGAGGGCAAGGTG	CTGGTTGCGGCCCTGCGC		
CCP2	Cre04.g222750	1065	ATGGCATCCGACGCTATGACTATCAACGAGG	CTGAGCGGTGGCGGGGCCGAT		
CAH1	Cre04.g223100	1131	ATGGCGCGTACTGGCGCTC	GTGATAGCGGGCCAGCTTGATG		
CAH3	Cre09.g415700	930	ATGCGCTCAGCCGTTCTAC	CAGCTCGTATTCGACCAGG		
CAH6	Cre12.g485050	792	ATGGGATGCGGTGCCAGCGTGC	CTCGGCGCTGCTGCCGCCCTT		
LCIA	synthetic codon optimised (Fig. S7)	1008	ATGCAGACAACTATGACAAGACCT	TGCTGTAGCAGGTTTAGCAGAT		
HLA3	synthetic codon optimised (Fig. S7)	3975	ATGGGAGAGGGGAAGGTG	TTGATTACGGCCTTGCG		
